# Termination of Resuscitation Rules and Survival Among Patients With Out-of-Hospital Cardiac Arrest: A Systematic Review and Meta-Analysis

**DOI:** 10.1001/jamanetworkopen.2024.20040

**Published:** 2024-07-03

**Authors:** Michael A. Smyth, Imogen Gunson, Alison Coppola, Samantha Johnson, Robert Greif, Kasper G. Lauridsen, Sian Taylor-Philips, Gavin D. Perkins

**Affiliations:** 1 Medical School, University of Warwick, Coventry, England; 2 University Hospital Coventry and Warwickshire NHS Trust, Coventry, England; 3 West Midlands Ambulance Service University NHS Foundation Trust, Brierly Hill, England; 4 University Hospitals Plymouth NHS Trust, Plymouth, England; 5 Department of Anesthesiology and Pain Therapy, Bern University Hospital, Inselspital, University of Bern, Bern, Switzerland; 6 School of Medicine, Sigmund Freud University, Vienna, Austria; 7 Research Center for Emergency Medicine, Aarhus University Hospital, Aarhus, Denmark; 8 Department of Medicine, Randers Regional Hospital, Randers, Denmark; 9 Department of Anesthesiology and Critical Care Medicine, Children’s Hospital of Philadelphia, Philadelphia, Pennsylvania; 10 University Hospitals Birmingham NHS Foundation Trust, Birmingham, England

## Abstract

**Importance:**

Termination of resuscitation (TOR) rules may help guide prehospital decisions to stop resuscitation, with potential effects on patient outcomes and health resource use. Rules with high sensitivity risk increasing inappropriate transport of nonsurvivors, while rules without excellent specificity risk missed survivors. Further examination of the performance of TOR rules in estimating survival of out-of-hospital cardiac arrest (OHCA) is needed.

**Objective:**

To determine whether TOR rules can accurately identify patients who will not survive an OHCA.

**Data Sources:**

For this systematic review and meta-analysis, the MEDLINE, Embase, CINAHL, Cochrane Library, and Web of Science databases were searched from database inception up to January 11, 2024. There were no restrictions on language, publication date, or time frame of the study.

**Study Selection:**

Two reviewers independently screened records, first by title and abstract and then by full text. Randomized clinical trials, case-control studies, cohort studies, cross-sectional studies, retrospective analyses, and modeling studies were included. Systematic reviews and meta-analyses were reviewed to identify primary studies. Studies predicting outcomes other than death, in-hospital studies, animal studies, and non–peer-reviewed studies were excluded.

**Data Extraction and Synthesis:**

Data were extracted by one reviewer and checked by a second. Two reviewers assessed risk of bias using the Revised Quality Assessment Tool for Diagnostic Accuracy Studies. Cochrane Screening and Diagnostic Tests Methods Group recommendations were followed when conducting a bivariate random-effects meta-analysis. This review followed the Preferred Reporting Items for a Systematic Review and Meta-Analysis of Diagnostic Test Accuracy Studies (PRISMA-DTA) statement and is registered with the International Prospective Register of Systematic Reviews (CRD42019131010).

**Main Outcomes and Measures:**

Sensitivity and specificity tables with 95% CIs and bivariate summary receiver operating characteristic (SROC) curves were produced. Estimates of effects at different prevalence levels were calculated. These estimates were used to evaluate the practical implications of TOR rule use at different prevalence levels.

**Results:**

This review included 43 nonrandomized studies published between 1993 and 2023, addressing 29 TOR rules and involving 1 125 587 cases. Fifteen studies reported the derivation of 20 TOR rules. Thirty-three studies reported external data validations of 17 TOR rules. Seven TOR rules had data to facilitate meta-analysis. One clinical study was identified. The universal termination of resuscitation rule had the best performance, with pooled sensitivity of 0.62 (95% CI, 0.54-0.71), pooled specificity of 0.88 (95% CI, 0.82-0.94), and a diagnostic odds ratio of 20.45 (95% CI, 13.15-31.83).

**Conclusions and Relevance:**

In this review, there was insufficient robust evidence to support widespread implementation of TOR rules in clinical practice. These findings suggest that adoption of TOR rules may lead to missed survivors and increased resource utilization.

## Key Points

**Question:**

Can termination of resuscitation (TOR) rules accurately identify patients who will not survive an out-of-hospital cardiac arrest?

**Findings:**

This systematic review and meta-analysis identified 43 studies describing the performance of TOR rules, but evidence concerning the ability of TOR rules to discriminate between those patients who will die and those who will survive was lacking. The available studies provided low-certainty evidence suggesting that the universal termination of resuscitation (UTOR) rule has the best performance; however, even the UTOR rule may not be suitable for use in systems in which transport rates are low and the survival rate is higher than 8%.

**Meaning:**

These findings suggest that there is insufficient robust evidence to support widespread implementation of TOR rules in clinical practice.

## Teaser

This systematic review and meta-analysis examines the use of termination of resuscitation rules to estimate patient survival of out-of-hospital cardiac arrest.

## Introduction

The incidence of out-of-hospital cardiac arrest (OHCA) in Europe is 67 to 170 per 100 000 inhabitants.^1^ Emergency medical services (EMS) personnel attempt resuscitation in 50% to 60% of cases and survival to discharge is 8% (range, 0%-18%).^1^ The decision to discontinue resuscitation is challenging. Influencing factors include decisional conflict,^2,3^ cardiac arrest location,^2,4^ medicolegal concerns,^2^ psychological comfort,^4^ experience,^4^ knowledge of survival outcomes,^4^ and education.^5^ Termination of resuscitation (TOR) rules have been developed to inform decision-making.^6,7,8^ These rules have the potential to affect patient outcomes and health resource use. Rules with poor specificity risk premature discontinuation of resuscitation. Rules with high sensitivity increase the number of futile transports and consume valuable health resources.

The International Liaison Committee on Resuscitation identified the evaluation of TOR rules for OHCA as a high priority.^9^ A review of in-hospital TOR rules was published previously.^10^ Therefore, this study sought to evaluate the performance of TOR rules in OHCA.

## Methods

This systematic review and meta-analysis followed the Preferred Reporting Items for a Systematic Review and Meta-Analysis of Diagnostic Test Accuracy Studies (PRISMA-DTA) statement. The protocol is registered with the International Prospective Register of Systematic Reviews (CRD42019131010).

We followed best practice recommendations for analyzing systematic reviews of diagnostic tests advocated by the Cochrane Screening and Diagnostic Tests Methods Group.^11^ We utilized test evaluation methods, rather than prognosis analysis methods, because these are easier to understand and there is precedent for using this approach.^12,13,14^ The reference standard (died or survived) is a dichotomous outcome that occurs soon after the index test (TOR rule prediction) is applied. Consequently, follow-up time for TOR rules is minimal. Unlike test evaluation methods, prognosis analysis methods include consideration of follow-up time and are therefore less appropriate for analysis of TOR rules.

### Eligibility Criteria

We included systematic reviews, meta-analyses, randomized clinical trials, case-control studies, cohort studies, cross-sectional studies, retrospective analyses, and modeling studies. Systematic reviews and meta-analyses were reviewed to identify primary studies. We excluded studies that predicted outcomes other than death or included only post–return of spontaneous circulation (ROSC) populations, non–peer-reviewed studies, in-hospital studies, and animal studies.

### Literature Search

We searched the MEDLINE, Embase, CINAHL, Cochrane Library, and Web of Science databases from inception up to January 11, 2024 (eTables 1-6 in Supplement 1). Reference lists were scrutinized, and subject area experts were contacted to identify missed studies. There were no restrictions on language, publication date, or time frame of the study.

### Study Selection

Duplicate records were removed. Screening occurred in 2 stages. First, 2 reviewers (I.G. and A.C.) independently reviewed each title and abstract and rated them as “include” or “exclude.” Any record rated as include by either reviewer was considered in stage 2. All other records were rejected as irrelevant. In stage 2, the reviewers (I.G. and A.C.) assessed the full text of the remaining records. Records rated as include by both reviewers were included, whereas those excluded by both reviewers were rejected as irrelevant. Where reviewers disagreed, this was resolved by consulting a third reviewer (M.A.S.).

### Data Extraction

Data were extracted using a predefined, piloted data extraction form by 1 reviewer (M.A.S.) and were checked by a second reviewer (either I.G. or A.C.). The data extraction form included study characteristics and contingency tables. If contingency data were not reported, they were calculated from sensitivity, specificity, and prevalence.

### Quality Assessment

Risk of bias was independently assessed by 2 reviewers (I.G. and A.C.) using either the ROBINS-I (Risk of Bias in Nonrandomised Studies of Interventions) tool^15^ or the Cochrane RoB 2 (Revised Risk of Bias for Randomized Trials) tool^16^ and the QUADAS-2 (Revised Quality Assessment Tool for Diagnostic Accuracy Studies) risk of bias and applicability concerns checklist.^17^ The Deeks funnel plot asymmetry test was used to identify publication bias. We did not calculate an *I*^2^ statistic because this is not recommended for systematic reviews of test accuracy.^18^ To assess heterogeneity, we assessed the symmetry of the summary receiver operating characteristic (SROC) curve and calculated the correlation coefficient.^19^ We adopted the Grading of Recommendations, Assessment, Development and Evaluation (GRADE) Working Group methodology to determine certainty of evidence.^20^

### Outcomes

For outcomes, we adopted Morrison’s^12^ recommendation to code death as the true positive. A true positive indicates stop resuscitation and the patient dies, a false positive indicates stop resuscitation but the patient survives (missed survivors), a true negative indicates continue resuscitation and the patient survives, and a false negative indicates continue resuscitation but the patient dies (futile resuscitations).

### Statistical Analysis

We analyzed derivation, external validation, and clinical studies separately. Derivation studies use regression methods and develop rules “trained” to the available dataset. External data validation studies evaluate TOR rules in a different dataset to assess generalizability, modeling ideal performance and avoiding the complexities introduced by clinician interaction. Clinical studies describe TOR rule performance in routine clinical practice. Meta-analysis of derivation, validation, and clinical studies together has the potential to bias estimates of TOR rule performance in clinical practice.

Statistical analysis was performed in R Studio, version 1.2.5042 (R Project for Statistical Computing),^21^ using several packages. Univariate analysis required contingency table data to calculate summary estimates using epiR, version 2.0.65.^22^ We used Meta, version 6.2-1,^23^ to generate the Deeks funnel plot. We conducted a bivariate random-effects meta-analysis using a generalized linear mixed model as advocated by Reitsma et al.^24^ We calculated the area under the curve (AUC) and produced bivariate SROC curves using Metafor, version 4.0-0.^19^ We calculated pooled sensitivity, specificity, and diagnostic odds ratios (DORs) using Meta, version 6.2-1.^23^ Estimates of effects at different prevalence levels were calculated using the GRADEPro Guideline Development Tool^25^ and were subsequently used to estimate the effects of TOR rules by calculating terminate and transport rates, miss rates, miss frequency, survivor rates, survivor frequency, and futile transport rates. Numbers of cases (in lieu of patients) are reported, because several studies used the same database, which meant patients could be counted more than once. Statistical significance was established at *P* < .05 (2-tailed).

## Results

**Figure 1.**
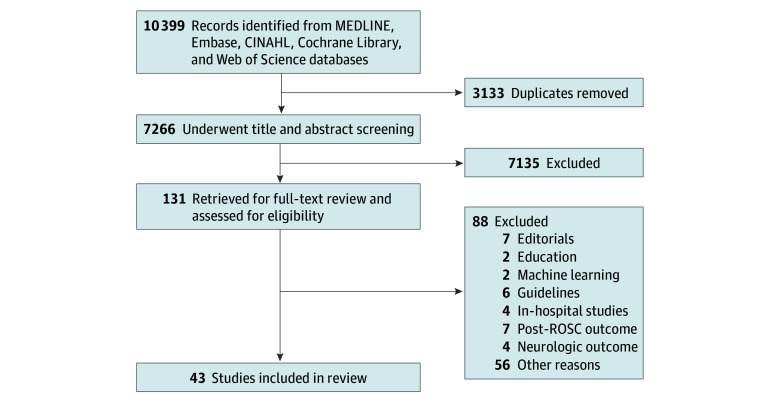
Study Flow Diagram ROSC indicates return of spontaneous circulation populations.

The database searches yielded 10 399 records. No additional studies were identified by searching reference lists or contacting subject experts. After deduplication, 7266 records remained. First-pass title and abstract screening yielded 131 potentially eligible studies. After the second-pass full-text screening, 43 studies^26,27,28,29,30,31,32,33,34,35,36,37,38,39,40,41,42,43,44,45,46,47,48,49,50,51,52,53,54,55,56,57,58,59,60,61,62,63,64,65,66,67,68^ remained (Figure 1).

### Study Characteristics

No randomized clinical trials were identified. This review included 43 nonrandomized studies involving 1 125 587 cases from 11 different countries. Publication dates spanned 1993 to 2023. There was substantial variation in study populations and prevalence across the included studies (eTables 7-9 in Supplement 1). Most studies reported death prior to hospital discharge; however, 1 study utilized death within 1 month.^36^ Three studies collected data prospectively,^30,37,55^ whereas the remainder utilized retrospective datasets. A summary of each study and a description of each TOR rule is provided in eTables 10 and 11, respectively, in Supplement 1.

Several studies have incorrectly reported efficacy of the basic life support (BLS) TOR rule. The BLS and UTOR rules use the same variables, but the BLS TOR rule captures use by BLS responders only.^65^ When the BLS TOR rule is applied within systems deploying clinicians operating above the BLS level, it should be reported as the UTOR rule.^54^ Multiple studies reporting BLS TOR rule performance included responders who operated above the BLS level.^27,33,36,41,42,46,47,58,60,64,68^ However, clinicians providing BLS cannot administer adrenaline or undertake advanced airway interventions. They therefore experience lower rates of ventricular fibrillation and return of spontaneous circulation (ROSC)—both variables of the BLS and UTOR rules. To accurately describe the performance of the BLS TOR and UTOR rules, we reclassified the aforementioned studies to facilitate accurate meta-analysis.

### Risk of Bias

Summary risk of bias and individual study risk of bias are reported in eFigures 1 and 2, respectively, in Supplement 1. There was substantial concern for patient selection, as many studies excluded subsections of the cardiac arrest population. Low risk of bias for the reference standard was expected due to the unambiguous nature of the outcome being assessed—the patient either lives or dies. These data are commonly reliable and readily confirmed. High risk of bias in flow and timing relates to concerns for verification bias; several studies were conducted in EMS systems practicing TOR, creating a self-fulfilling prophecy.

### Publication Bias

We generated a Deeks funnel plot for each TOR rule with 4 or more studies (the minimum required for computation).^69^ Asymmetry in the plot indicates potential publication bias.^69^ The generated plots (eFigure 3 in Supplement 1) suggested no concern for publication bias for the advanced life support (ALS) and UTOR rules. Publication bias may be a concern for the BLS rule; however, this was uncertain because there were few included studies. Insufficient data prevented generation of Deeks plots for the Marsden, Petrie, Goto 1, and Shibahashi 1 TOR rules.

### Derivation Studies

We identified very low-certainty evidence (downgraded for risk of bias, inconsistency, imprecision, and indirectness) from 15 observational studies^26,29,34,36,40,41,44,49,51,53,59,64,65,68,70^ involving 198 442 cases. These studies reported the derivation of 20 unique TOR rules. eTable 7 in Supplement 1 presents their sensitivity and specificity with 95% CIs. Because each derivation rule is distinct, meta-analysis is not appropriate and no heterogeneity assessment was undertaken.

### External Validation Studies

We identified very low-certainty evidence (downgraded for risk of bias, inconsistency, imprecision, indirectness, and publication bias) from 33 observational studies^27,28,30,31,32,33,35,36,37,38,39,41,42,43,45,46,47,48,49,50,52,53,54,56,57,58,60,62,63,64,66,67,68^ involving 927 534 cases. These studies reported external data validations of 17 TOR rules. eTable 8 in Supplement 1 presents their sensitivity and specificity with 95% CIs. Within these 33 studies, we identified 7 TOR rules (BLS, ALS, UTOR, Marsden, Petrie, Shibahashi 1, and Goto 1) for which meta-analysis was possible.

**Figure 2.**
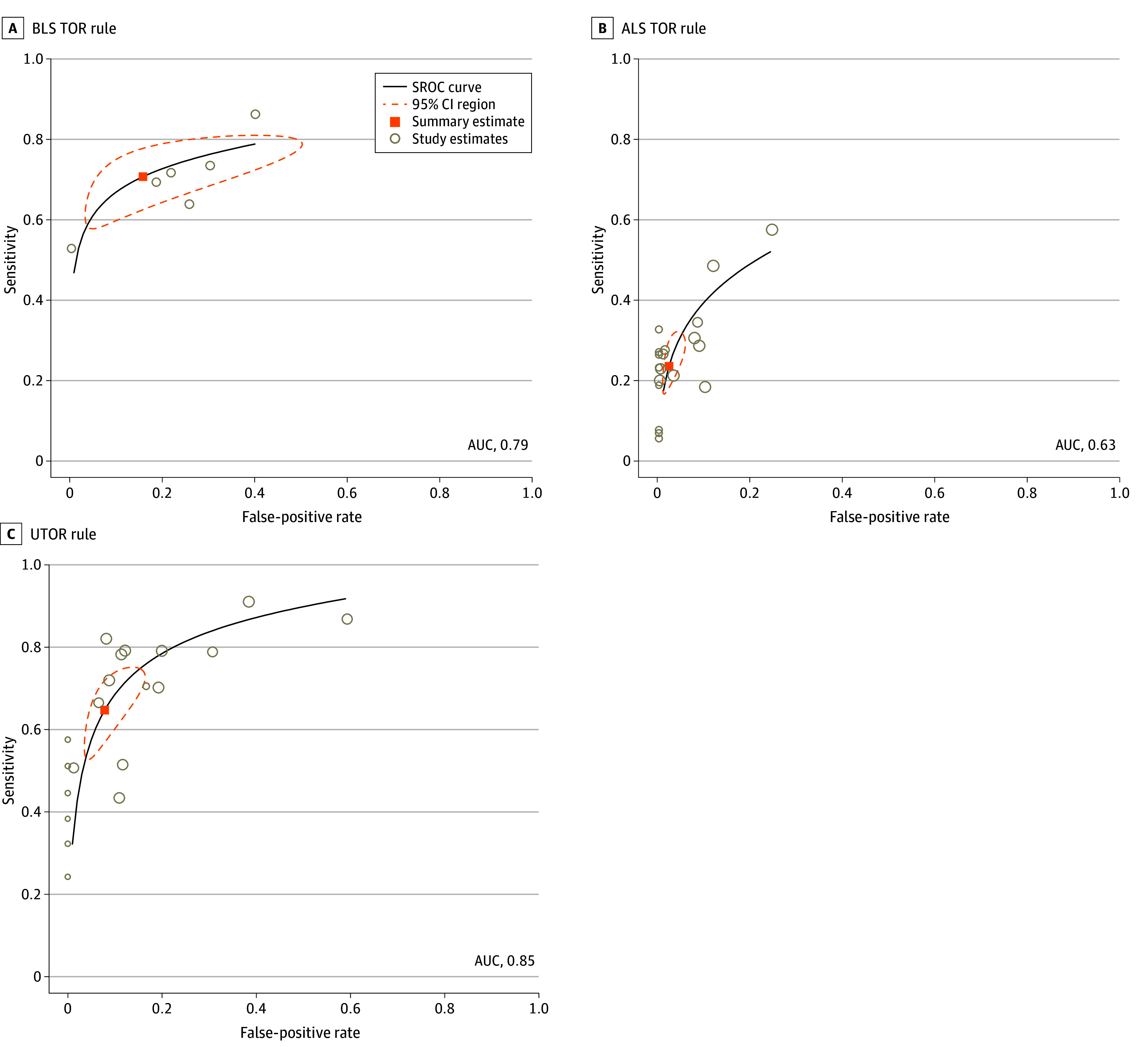
Bivariate Summary Receiver Operating Characteristic (SROC) Curves ALS indicates advanced life support; AUC, area under the curve; BLS, basic life support; UTOR, universal termination of resuscitation.

Bivariate SROC curves are shown in Figure 2 for the BLS, ALS, and UTOR rules and in eFigure 4 in Supplement 1 for the remaining rules. Correlation coefficients for the BLS, ALS, and UTOR rules were −0.77, −0.65, and −0.72, respectively, suggesting that heterogeneity between studies was not a notable concern. It was not possible to reliably estimate correlation coefficients for the Marsden, Petrie, Goto 1, and Shibahashi 1 TOR rules because there were insufficient studies. Our meta-analysis suggests that the Petrie rule performed poorly (AUC, 0.56), whereas the ALS rule failed to reach acceptable standards (AUC, 0.63). Both the BLS (AUC, 0.79) and Shibahashi 1 (AUC, 0.75) rules achieved acceptable performance, whereas the UTOR (AUC, 0.85), Marsden (AUC, 0.81), and Goto 1 (AUC, 0.85) rules had excellent performance (Figure 2 and eFigure 4 in Supplement 1). Pooled estimates of effect are reported in Table 1, a summary of findings with estimates of effect is reported in Table 2, and estimated performance at different prevalence rates is reported in Table 3.

**Table 1.** Pooled Summary Estimates for External Data Validation Studies

TOR rule	No. of studies	Sensitivity (95% CI)	Specificity (95% CI)	DOR (95% CI)
BLS	6	0.66 (0.59-0.74)	0.81 (0.70-0.91)	13.70 (3.76-49.83)
ALS	17	0.27 (0.20-0.34)	0.96 (0.93-0.99)	9.21 (5.77-14.69)
UTOR	19	0.63 (0.54-0.72)	0.88 (0.81-0.94)	21.86 (13.81-34.60)
Marsden	2	0.42 (0-0.86)	0.97 (0.88-1.00)	44.97 (5.54-365.23)
Petrie	2	0.21 (0-0.43)	0.98 (0.93-1.00)	20.05 (2.14-187.57)
Goto 1	2	0.46 (0.32-0.61)	0.93 (0.90-0.97)	12.33 (10.96-13.86)
Shibahashi 1	2	0.30 (0.17-0.41)	0.96 (0.93-0.99)	9.51 (7.70-11.75)

Abbreviations: ALS, advanced life support; BLS, basic life support; DOR, diagnostic odds ratio; UTOR, universal termination of resuscitation.

**Table 2.** Summary of Findings for Estimates of Effect for TOR Rules at Different Survival Rates^a^

TOR rule	No. of studies (cases)^b^	Factors that may decrease certainty of evidence					Certainty of evidence	Outcome	Estimate of effect per 1000 patients (95% CI)			Pooled sensitivity, pooled specificity, and survival range^c^
		Risk of bias	Indirectness	Inconsistency	Imprecision	Other			8% Survival	10% Survival	12% Survival	
BLS	6 (36 325)	Very serious	Very serious	Serious	Not serious	Publication bias	Very low	TP	641 (559-722)	627 (547-707)	613 (535-691)	0.66 (0.59-0.74), 0.81 (0.70-0.91), 1.4-14.7
								FP	18 (10-27)	23 (12-34)	28 (14-41)	
								FN	279 (198-361)	273 (193-353)	267 (189-345)	
								TN	62 (53-70)	77 (66-88)	92 (79-106)	
UTOR	19 (369 631)	Very serious	Very serious	Serious	Not serious	Publication bias	Very low	TP	574 (497-652)	562 (486-638)	549 (475-624)	0.62 (0.54-0.71), 0.88 (0.81-0.94), 1.4-19.9
								FP	10 (5-15)	12 (6-19)	15 (7-22)	
								FN	346 (268-423)	338 (262-414)	331 (256-405)	
								TN	70 (65-75)	88 (81-94)	105 (98-113)	
ALS	17 (276 861)	Very serious	Very serious	Serious	Not serious	Publication bias	Very low	TP	236 (183-290)	231 (179-283)	226 (175-277)	0.26 (0.20-0.31), 0.96 (0.93-0.99), 1.2-15.6
								FP	3 (1-5)	4 (1-7)	5 (1-8)	
								FN	684 (630-737)	669 (617-721)	654 (603-705)	
								TN	77 (75-79)	96 (93-99)	115 (112 to 119)	
Marsden	2 (15 953)	Very serious	Very serious	Serious	Not serious	Publication bias	Very low	TP	387 (0-795)	378 (0-778)	370 (0-760)	0.42 (0.0-0.86), 0.97 (0.89-1.0), 1.4-4.6
								FP	3 (0-9)	3 (0-11)	4 (0-13)	
								FN	533 (125-920)	522 (122-900)	510 (120-880)	
								TN	77 (71-80)	97 (89-100)	116 (107-120)	
Petrie	2 (15 953)	Very serious	Very serious	Serious	Not serious	Publication bias	Very low	TP	193 (0-392)	188 (0-383)	184 (0-375)	0.21 (0.0-0.43), 0.98 (0.93-1.0), 1.4-4.6
								FP	1 (0-6)	2 (0-7)	2 (0-8)	
								FN	727 (528-920)	712 (517-900)	696 (505-880)	
								TN	79 (74-80)	98 (93-100)	118 (112-120)	
Goto 1	2 (59 672)	Very serious	Very serious	Serious	Not serious	Publication bias	Very low	TP	426 (291-561)	417 (284-549)	408 (278-537)	0.46 (0.32-0.61), 0.93 (0.90-0.97), 6.0-11.7
								FP	6 (3-8)	7 (3-10)	8 (4-13)	
								FN	494 (359-629)	483 (351-616)	472 (343-602)	
								TN	74 (72-77)	93 (90-97)	112 (107-116)	
Shibahashi 1	2 (80 939)	Very serious	Very serious	Serious	Not serious	Publication bias	Very low	TP	266 (158-375)	261 (154-367)	255 (151-359)	0.29 (0.17-0.41), 0.96 (0.93-0.99), 6.0%-9.3%
								FP	3 (1-6)	4 (1-7)	5 (1-9)	
								FN	654 (545-762)	639 (533-746)	625 (521-729)	
								TN	77 (74-79)	96 (93-99)	115 (111-119)	

Abbreviations: ALS, advanced life support; BLS, basic life support; FN, false negative; FP, false positive; TN, true negative; TOR, termination of resuscitation; TP, true positive; UTOR, universal termination of resuscitation.

^a^ Included studies met the following criteria: (1) population: patients experiencing out-of-hospital cardiac arrest; (2) index test: TOR rule recommending termination of resuscitation; (3) reference standard: true outcome (patient died or survived); and (4) study type: external validation studies of TOR rules.

^b^ All were nonrandomized.

^c^ Pooled sensitivity and specificity values are presented with 95% CIs. Percent survival is presented as a range.

**Table 3.** Estimated Performance of TOR Rule Use at Different Prevalence Rates^a^

TOR rule, % survival	Discontinue, %^b^	Discontinue decision, %^c^		Miss, %^d^	Miss frequency^e^	Transport, %^f^	Transport decision, %^g^		Survivor, %^h^	Survivor frequency^i^	Futile transport, %^j^
		Correct	Incorrect				Correct	Incorrect			
BLS											
8	65.9 (63.5-69.4)	97.3 (96.4-98.2)	2.7 (1.8-3.6)	1.8 (1.2-2.3)	56 (44-82)	34.1 (30.6-36.5)	18.2 (16.2-21.1)	81.8 (78.9-83.8)	6.2 (5.9-6.5)	16.1 (15.5-16.9)	28 (24-31)
10	65.0 (62.7-68.3)	96.5 (95.4-97.9)	3.5 (2.1-4.6)	2.3 (1.5-2.9)	44 (35-68)	35.0 (31.7-37.3)	22.0 (20.0-25.5)	78.0 (74.5-80.0)	7.7 (7.4-8.1)	13.0 (12.4-13.4)	27 (24-30)
12	64.1 (61.9-67.7)	95.6 (94.4-97.4)	4.4 (2.6-5.6)	2.8 (1.7-3.5)	36 (29-58)	35.9 (32.8-38.1)	25.6 (23.5-29.5)	74.4 (70.5-76.5)	9.2 (9.0-9.7)	10.9 (10.3-11.2)	27 (23-29)
UTOR											
8	58.4 (57.3-60.1)	98.3(97.8-99.0)	1.7 (1.0-2.2)	1.0 (0.6-1.3)	100 (167-78)	41.6 (39.9-42.7)	16.8 (15.1-19.5)	83.2 (80.5-84.9)	7.0 (6.4-7.8)	14 (13-16)	35 (32-36)
10	57.4 (56.4-58.9)	97.9 (97.1-98.8)	2.1 (1.2-2.9)	1.2 (0.7-1.6)	83 (61-139)	42.6 (41.1-43.6)	20.7 (18.5-23.6)	79.3 (76.4-81.5)	8.8 (8.1-9.7)	11 (10-12)	34 (31-36)
12	56.4 (55.5-57.7)	97.3 (96.6-98.5)	2.7 (1.5-3.4)	1.5 (0.8-1.9)	67 (53-119)	43.6 (42.3-44.5)	24.1 (21.8-27.7)	75.9 (72.3-78.2)	10.5 (9.7-11.7)	9.5 (8.5-10.3)	33 (31-35)
ALS											
8	23.9 (20.7-26.6)	98.7 (98.3-99.5)	1.3 (0.5-1.7)	0.3 (0.1-0.5)	333 (222-889)	76.1 (73.4-79.3)	10.1 (9.7-10.6)	89.8 (89.4-90.3)	7.7 (7.1-8.4)	13 (12-14)	68 (66-71)
10	23.5 (20.2-23.5)	98.3 (97.6-99.4)	1.7 (0.6-2.4)	0.4 (0.1-0.6)	250 (159-890)	76.5 (73.9-79.8)	12.5 (12.1-13.1)	87.5 (86.9-87.9)	9.6 (8.9-10.4)	10.4 (9.6-11.2)	67 (65-69)
12	23.1 (19.8-25.7)	97.8 (97.2-99.4)	2.2 (0.6-2.8)	0.5 (0.1-0.7)	22 (139-891)	76.9 (74.3-80.2)	15.0 (14.4-15.7)	85.0 (84.3-85.6)	11.5 (10.7-12.6)	8.7 (8.0-9.3)	65 (64-68)
Marsden											
8	39.0 (0.1-44.6)	99.2 (50.0-98.9)	0.8 (1.1-50.0)	0.3 (0.1-0.5)	333 (200-1962)	61.0 (55.4-99.9)	12.6 (8.0-36.2)	87.4 (63.8-92.0)	7.7 (4.4-36.2)	13.0 (2.8-22.6)	53 (51-64)
10	38.1 (0.1-44.1)	99.2 (98.9-1.0)	0.8 (0-1.1)	0.3 (0-0.6)	333 (163-2111)	61.9 (55.9-100)	15.7 (10.0-42.2)	84.3 (57.8-90.0)	9.7 (5.6-42.2)	10.3 (2.4-17.9)	52 (50-58)
12	37.4 (0.1-43.6)	98.9 (98.3-1.0)	1.1 (0-1.7)	0.4 (0-0.7)	250 (136-2271)	62.6 (56.4-100)	18.5 (12.0-47.1)	81.5 (52.9-88.0)	11.6 (6.8-47.1)	8.6 (2.1-14.8)	51 (50-53)
Petrie											
8	19.4 (0-28.5)	99.5 (98.5-1.0)	0.5 (0-1.5)	0.1 (0-0.4)	1000(233-5811)	80.6 (71.5-100)	9.8 (8.0-12.3)	90.2 (92.0-87.7)	7.9 (5.7-12.3)	12.7 (8.1-17.5)	73 (66-88)
10	19.0 (0-28.1)	98.9 (98.2-1.0)	1.1 (0-1.8)	0.2 (0-0.5)	500 (198-6102)	81.0 (71.9-100)	12.1 (10.0-15.2)	87.9 (84.8-90.0)	9.8 (7.2-15.2)	10.2 (6.6-13.9)	71 (65-85)
12	18.6 (0-27.7)	98.9 (97.9-1.0)	1.1 (0-2.1)	0.2 (0-0.6)	500 (173-6171)	81.4 (72.3-100)	14.5 (12.0-18.2)	85.5 (81.8-88.0)	11.8 (8.7-18.1)	8.5 (5.5-11.5)	70 (64-82)
Goto 1											
8	43.2 (40.6-44.6)	98.6 (98.6-99.0)	1.4 (1.0-1.4)	0.6 (0.4-0.6)	167 (159-242)	56.8 (55.4-59.4)	13.0 (10.9-16.7)	87.0 (83.3-89.1)	7.4 (6.0-9.9)	13.5 (10.1-16.6)	49 (49-50)
10	42.4 (39.4-43.9)	98.3 (98.2-99.0)	1.7 (1.0-1.8)	0.7 (0.4-0.8)	143 (127-243)	57.6 (56.1-60.6)	16.1 (13.6-20.4)	83.9 (79.6-86.4)	9.3 (7.6-12.4)	10.8 (8.1-13.1)	48 (48-48)
12	41.6 (38.5-43.4)	98.1 (97.6-98.6)	1.9 (1.4-2.4)	0.8 (0.5-0.8)	125 (98-183)	58.4 (56.6-61.5)	19.2 (16.2-23.8)	80.8 (76.2-83.8)	11.2 (9.1-14.6)	8.9 (6.8-10.9)	47 (47-47)
Shibahashi 1											
8	26.9 (20.4-31.2)	98.9 (98.4-99.4)	1.1 (0.6-1.6)	0.3 (0.1-0.5)	333 (204-778)	73.1 (68.8-79.6)	10.5 (9.4-12.0)	89.5 (88.0-90.6)	7.7 (6.5-9.5)	13.0 (10.5-15.5)	65 (62-70)
10	26.5 (19.8-30.7)	98.5 (98.1-99.4)	1.5 (0.6-1.9)	0.4 (0.1-0.6)	250 (174-781)	73.5 (69.3-80.2)	13.1 (11.7-14.9)	86.9 (85.1-88.3)	9.6 (8.1-11.9)	10.4 (8.4-12.3)	64 (61-68)
12	26.0 (19.4-30.3)	98.1 (97.6-99.3)	1.9 (0.7-2.4)	0.5 (0.1-0.7)	200 (135-784)	74.0 (69.7-80.6)	15.5 (14.0-17.6)	88.3 (82.4-84.5)	11.5 (9.8-14.2)	8.7 (7.1-10.2)	63 (60-66)

Abbreviations: ALS, advanced life support; BLS, basic life support; FN, false negative; FP, false positive; TN, true negative; TOR, termination of resuscitation; TP, true positive; UTOR, universal termination of resuscitation.

^a^ Values are presented with 95% CIs.

^b^ Calculated as [(TP + FP)/(TP + FP + FN + TN)].

^c^ Calculated as [(TP)/(TP + FP)] for correct and [(FP)/(TP + FP)] for incorrect.

^d^ Calculated as [(FP)/(TP + FP + FN + TN)].

^e^ Calculated as [1/(% Miss)].

^f^ Calculated as [(TN + FN)/(TP + FP + FN + TN)].

^g^ Calculated as correct – [(TN)/(FN + TN)] for correct and [(FN)/(FN + TN)] for incorrect.

^h^ Calculated as [(TN)/(TP + FP + FN + TN)].

^i^ Calculated as [1/(% Survivor)].

^j^ Calculated as [(FN)/(TP + FP + FN + TN)].

### Clinical Studies

We identified very low-certainty evidence (downgraded for indirectness) from 1 Canadian study^55^ involving 954 cases (eTable 9 in Supplement 1). This study described the clinical validation of the BLS rule.^55^ The study had sensitivity of 0.64 (95% CI, 0.61-0.68), specificity of 1.00 (95% CI, 0.92-1.00), and positive predictive value of 1.00 (95% CI, 0.99-1.00). The BLS rule recommended transport for 367 of 953 cases (38.5%); of these 367, 44 (12.0%) survived to discharge and 323 (88.0%) died in the hospital. The BLS rule recommended TOR for the remaining 586 cases; however, resuscitation was terminated for only 388 (66.2%). Ambulance crews transported 198 patients to the hospital despite the recommendation of the BLS rule to stop resuscitation; none of these patients survived.

## Discussion

To our knowledge, this review is the first to analyze studies by derivation, external data validation, and clinical categories to minimize the bias that may be introduced by pooling these categories. This meta-analysis is also the first, to our knowledge, to reclassify studies incorrectly reporting the efficacy of the BLS rule rather than the UTOR rule, enabling an accurate performance assessment of these 2 TOR rules by their intended clinician populations. Finally, our study is the only meta-analysis to date to estimate TOR rule performance at different prevalence levels (88%, 90%, and 92% [12%, 10%, and 8% survival]).

The TOR rules are intended to differentiate between those patients for whom resuscitation can be safely discontinued and those who might benefit from further (hospital) treatment.^26^ Traditionally reported metrics to describe TOR rule performance include sensitivity, specificity, positive predictive value, transport rate, and miss rate. These metrics frame the performance of the TOR rule with respect to patient safety (how many potential survivors are missed) and resource utilization (reduced number of hospital transports).

Early TOR rule studies reported 100% specificity. However, it may be unrealistic to expect a TOR rule with 100% specificity that does not miss any potential survivors.^12^ This argument asserts that the ethically acceptable threshold for medical futility is 1% and that specificity of 99% (a miss rate of 1%) should therefore represent acceptable TOR rule performance.^71^ Conversely, the European Resuscitation Council argued that success rates of less than 1% still justify a resuscitation effort, questioning the acceptability of a 1% miss rate.^72^ Our analysis suggests that miss rates could range from 0.1% (95% CI, 0%-0.4%) for the Petrie rule to 1.8% (95% CI, 1.2%-2.3%) for the BLS rule (assuming 8% survival; Table 3).

Studies on TOR rules frequently report statistically significant reductions in transport rates. However, these estimates seldom reflect practice in Western EMS systems. Verhaert et al^66^ reported that the ALS rule recommended transport for 94% of cases in a system that only transported 54% of cases. The most recent data from English ambulance services show that 41.7% of cases were transported to the hospital.^73^ Our analysis suggests that transport rates would vary from 34.1% (range, 30.6%-36.5%) for the BLS rule to 80.6% (range, 71.5%-100%) for the Petrie rule when prevalence is 8% (Table 3). Lower transport rates most likely occur because clinical practice guidelines provide additional scope not to start resuscitation in cases in which it would be futile, for patients with terminal illness, or where the patient has expressed a wish not to be resuscitated. Current TOR rules lack this flexibility.

Of the TOR rules identified in our meta-analysis, the BLS rule could not be implemented in the UK because the EMS system utilizes ALS-level paramedics. Neither the Goto 1 and Shibahashi 1 TOR rules could be implemented legally because they both discriminate by age and would contravene the UK Equality Act of 2010.^74^ The ALS, Goto 1, and Marsden TOR rules include bystander cardiopulmonary resuscitation (CPR) as a variable, requiring continued resuscitation (eTable 11 in Supplement 1). Currently, bystander CPR rates in England approach 70%, suggesting that these rules may be less helpful in a UK context because they will automatically recommend that the majority of patients be transported. Of the remaining TOR rules examined in this review, the Petrie TOR rule had the best specificity but poor sensitivity. We found that the UTOR rule had the best sensitivity and DOR (Table 1). Based on both our AUC and DOR data, the UTOR rule had the best performance.

Our analysis suggests that the UTOR rule would miss 1.0% (95% CI, 0.6%-1.3%) of survivors (8% survival; Table 3). This means that 1 survivor would be missed for every 100 (95% CI, 78-167) resuscitation attempts. Paramedic exposure to cardiac arrest is low (range, 2-5 cardiac arrests per year).^75,76,77^ If a paramedic attended 3 cardiac arrests per year, then each paramedic would miss a survivor every 33.3 years. However, if survival were to improve to 10% (90% prevalence), then the miss rate would increase to 1.2% (higher than the “acceptable” 1% miss rate^71^), equating to an additional 68 missed survivors annually in England (based on 2021 data).^73^ Similarly, at 12% survival (88% prevalence), the miss rate would rise to 1.5%, equating to 170 missed survivors. In the context of only 2700 cardiac survivors nationally, the number of missed survivors is not insignificant and is unlikely to be deemed acceptable.

Unfortunately, common TOR rule metrics also overlook incorrect (false-negative) recommendations to continue resuscitation. This may be driven by a belief that continuing resuscitation to the hospital is not harmful. However, recent data indicate that transporting patients during resuscitation is associated with reduced probability of survival compared with resuscitation on scene.^78^ Furthermore, Wampler et al^79^ reported that survival was rare where ROSC was not achieved before initiating transport. International guidelines now recommend against routine transportation to the hospital unless needed to access treatment that EMS cannot provide on scene or when legal or cultural considerations mandate transfer.^80^ Transporting patients for whom resuscitation is futile is not benign: it consumes scarce emergency department resources and increases risk for ambulance clinicians. Up to 81.4% of work-related injuries among ambulance staff have been attributed to ambulance collisions.^81,82^ Compared with vehicles of a similar size, ambulance collisions occur more frequently,^83^ involve a greater number of casualties, and are more likely to result in substantial injury.^84^

Our findings suggest that in addition to mitigating risk, reducing futile transport will also realize financial benefits. At 8% survival, the UTOR rule recommends futile transport in 35% (95% CI, 32%-36%) of cases (Table 3). Recent data indicate that this equates to 11 900 futile transports in English EMS systems annually.^73^ Cost-effectiveness data from the PARAMeDIC2 trial suggested that death at the scene was associated with mean (SD) ambulance costs of £1793.89 (£1056.61) (US $2279.63 [$1342.71]), whereas transported patients who died within 24 hours had associated mean (SD) ambulance service costs of £1507.69 (£562.56) (US $1915.93 [$714.89]) and hospital costs of £682.44 (£1515.93) (US $867.23 [$1926.41]).^85^ This finding implies an incremental cost of £396.24 (US $503.53) associated with death following transport. Minimizing futile transport rates therefore has the potential to realize substantial savings. In England, the UTOR rule would incur potentially avoidable costs of £4.7 million (95% CI, £4.3-4.9 million) (approximately US $5.10 million [95% CI, US $5.5-6.2 million]), assuming 34 000 resuscitation attempts each year (assuming 8% survival).^73^

### Limitations

This review has the following limitations. All included studies were observational in design, and the majority were retrospective in nature. This limitation has important implications for the quality of the evidence and inferences that can be made from these data. We excluded studies of TOR rules predicting favorable neurologic outcomes rather than death, because patients, families, and communities place different value on survival with poor neurological outcome. Furthermore, estimation of neurological outcome at discharge or 30 days is unreliable due to improvements seen in postresuscitation care.^86^ Rigorous synthesis of the literature was further limited by heterogeneity in the populations studied, differences in the scope of practice of EMS personnel, diverse EMS system design, substantial variation in the quality of CPR and the prevalence of resuscitation outcomes, differences in how decisions are made to cease resuscitation, and the inherent risk of TOR rules creating a self-fulfilling prophecy.

## Conclusions

The findings of this systematic review and meta-analysis suggest that there is very low-certainty evidence concerning the ability of TOR rules to discriminate between patients who will die and those who will survive. The literature comprises mainly derivation and external data validation studies. Clinical studies are almost nonexistent. Our findings suggest that TOR rules may miss substantial numbers of survivors. In addition, futile transport is not consistent with evidence-based practice; it reduces the likelihood of survival, increases risk, consumes scarce emergency department resources, and incurs substantial avoidable costs. Therefore, we suggest that there is an urgent need to review the role of TOR rules.

## Notes

Supplement 1. **eTable 1.** PICOST Criteria **eTable 2.** MEDLINE Search Strategy **eTable 3.** Embase Search Strategy **eTable 4.** CINAHL Search Strategy **eTable 5.** Cochrane Search Strategy **eTable 6.** Web of Science Search Strategy **eTable 7.** Derivation Studies Paired Sensitivity and Specificity **eTable 8.** External Validation Studies Paired Sensitivity and Specificity **eTable 9.** Clinical Studies Paired Sensitivity and Specificity **eTable 10.** Summary of Included Studies **eTable 11.** Description of Termination of Resuscitation (TOR) Rules **eFigure 1.** QUADAS-2 Summary Risk of Bias and Applicability **eFigure 2.** Study Risk of Bias **eFigure 3.** Deeks Funnel Plot Asymmetry Test **eFigure 4.** Bivariate Summary Receiver Operating Characteristic (SROC) Curves
eReferences

Supplement 2.
Data Sharing Statement
