# Supplementary material for: Termination of Resuscitation Rules and Survival Among Patients With Out-of-Hospital Cardiac Arrest: A Systematic Review and Meta-Analysis
Source: JAMA Netw Open. 2024 Jul 3;7(7):e2420040. doi: 10.1001/jamanetworkopen.2024.20040 (PMC11222995; doi:10.1001/jamanetworkopen.2024.20040)
Supplement: Supplement 1. — eTable 1. PICOST Criteria eTable 2. MEDLINE Search Strategy eTable 3. Embase Search Strategy eTable 4. CINAHL Search Strategy eTable 5. Cochrane Search Strategy eTable 6. Web of Science Search Strategy eTable 7. Derivation Studies Paired Sensitivity and Specificity eTable 8. External Validation Studies Paired Sensitivity and Specificity eTable 9. Clinical Studies Paired Sensitivity and Specificity eTable 10. Summary of Included Studies eTable 11. Description of Termination of Resuscitation (TOR) Rules eFigure 1. QUADAS-2 Summary Risk of Bias and Applicability eFigure 2. Study Risk of Bias eFigure 3. Deeks Funnel Plot Asymmetry Test eFigure 4. Bivariate Summary Receiver Operating Characteristic (SROC) Curves eReferences [file jamanetwopen-e2420040-s001.pdf]

Supplementary Online Content

Smyth MA, Gunson I, Coppola A, et al. Termination of resuscitation rules and survival among patients with out-of-hospital cardiac arrest: a systematic review and meta-analysis. *JAMA Netw Open*. 2024;7(7):e2420040. doi:10.1001/jamanetworkopen.2024.20040

eTable 1. PICOST Criteria
eTable 2. MEDLINE Search Strategy
eTable 3. Embase Search Strategy
eTable 4. CINAHL Search Strategy
eTable 5. Cochrane Search Strategy
eTable 6. Web of Science Search Strategy
eTable 7. Derivation Studies Paired Sensitivity and Specificity
eTable 8. External Validation Studies Paired Sensitivity and Specificity
eTable 9. Clinical Studies Paired Sensitivity and Specificity
eTable 10. Summary of Included Studies
eTable 11. Description of Termination of Resuscitation (TOR) Rules
eFigure 1. QUADAS-2 Summary Risk of Bias and Applicability
eFigure 2. Study Risk of Bias
eFigure 3. Deeks Funnel Plot Asymmetry Test
eFigure 4. Bivariate Summary Receiver Operating Characteristic (SROC) Curves
eReferences

This supplementary material has been provided by the authors to give readers additional information about their work.

© 2024 Smyth MA et al. *JAMA Network Open.*

eTable 1. PICOST Criteria

Population:	Adults and children with out-of-hospital cardiac arrest, where resuscitation is attempted, but return of spontaneous circulation (ROSC) is not achieved.
Intervention:	Termination of resuscitation (TOR) rule predicted outcome.
Comparison:	True outcome
Outcomes:	Ability of TOR rule to predict death.<br>Economic implications of application of TOR rules.
Study Design:	*Inclusion criteria:* Systematic reviews, meta-analyses, randomised controlled trials, case-control studies, cohort studies, cross-sectional studies, retrospective analyses, economic evaluations, modelling studies.<br>*Exclusion criteria:* Non-peer reviewed studies (e.g., conference abstracts, trial protocols, narrative reviews, letters, editorials, commentaries), animal studies.<br>No language restrictions were placed.<br>We excluded studies predicting poor neurologic outcome rather than death. Although favourable neurologic outcome is critically important to patients, we were unable to identify any EMS systems that advocated termination of resuscitation where poor neurologic outcome was predicted.<br>We excluded studies addressing outcome for post-ROSC patients. Although it would be possible to transpose the data such that death becomes the true positive, the populations included in such studies comprise only ROSC patients, thus they are different to the cardiac arrest population.
Timeframe:	The initial search was conducted from database inception to mid-July 2019. Covid-19 resulted in the project being paused. The final update occurred in January 2024.

© 2024 Smyth MA et al. *JAMA Network Open.*

eTable 2. MEDLINE Search Strategy

1 exp Out-of-Hospital Cardiac Arrest/
2 ohca.mp.
3 exp Ventricular Fibrillation/ or exp Tachycardia, Ventricular/
4 heart arrest.mp. or exp Heart Arrest/
5 cardiopulmonary arrest.mp.
6 circulatory arrest.mp.
7 cardiac standstill.mp.
8 pulseless electrical activity.mp.
9 pea.mp.
10 pulseless.mp.
11 shockable.mp.
12 (non-shockable or non shockable).mp.
13 cardiac arrest.mp.
14 ventricular fibrillation.mp.
15 ventricular tachycardia.mp.
16 3 or 4 or 5 or 6 or 7 or 8 or 9 or 10 or 11 or 12 or 13 or 14 or 15
17 (out of hospital or out-of-hospital or prehospital or pre-hospital or pre hospital).mp.
18 exp Emergency Medical Services/ or emergency medical service*.mp.
19 exp Emergency Medical Technicians/ or paramedic*.mp.
20 ambulance*.mp. or exp Ambulances/
21 exp air ambulances/ or air ambulance*.mp. or hems.mp.
22 (ems or emt).mp.
23 field.mp.
24 17 or 18 or 19 or 20 or 21 or 22 or 23
25 16 and 24
26 1 or 2 or 25
27 exp Resuscitation Orders/ or exp Resuscitation/ or resuscitat*.mp.
28 cardiopulmonary resuscitation.mp. or exp Cardiopulmonary Resuscitation/
29 cpr.mp.
30 basic life support.mp. or exp Life Support Care/
31 exp Advanced Cardiac Life Support/ or advanced life support.mp.
32 (bls or als).mp.
33 exp Heart Massage/ or chest compression*.mp.
34 27 or 28 or 29 or 30 or 31 or 32 or 33
35 26 and 34
36 (terminat* or cease or cessation or stop or withdraw* or withhold* or withheld or TOR or futile or futility or rule* or algorithm* or decease* or decision* or prognosis or predict*).mp.
37 exp Prognosis/
38 36 or 37
39 35 and 38
40 (letter or case reports or editorial or lecture).pt.
41 39 not 40
42 limit 41 to humans

© 2024 Smyth MA et al. *JAMA Network Open.*

eTable 3. Embase Search Strategy

1 exp "out of hospital cardiac arrest"/
2 ohca.mp.
3 ventricular fibrillation.mp. or exp heart ventricle fibrillation/
4 ventricular tachycardia.mp. or exp heart ventricle tachycardia/
5 heart arrest.mp. or exp heart arrest/
6 exp cardiopulmonary arrest/ or cardiopulmonary arrest.mp.
7 circulatory arrest.mp.
8 cardiac standstill.mp.
9 pulseless electrical activity.mp.
10 pea.mp.
11 pulseless.mp.
12 shockable.mp.
13 (non-shockable or non shockable).mp.
14 cardiac arrest.mp.
15 or/3-14
16 (out of hospital or out-of-hospital or prehospital or pre-hospital or pre hospital).mp.
17 exp emergency health service/ or emergency medical service*.mp.
18 paramedic*.mp. or exp paramedical personnel/
19 emergency medical technician.mp. or exp rescue personnel/
20 exp air medical transport/ or air ambulance*.mp. or HEMS.mp.
21 exp ambulance/ or ambulance*.mp.
22 (ems or emt).mp.
23 field.mp.
24 or/16-22
25 15 and 24
26 1 or 2 or 25
27 exp resuscitation/ or resuscitat*.mp.
28 cardiopulmonary resuscitation.mp.
29 resuscitation orders.mp.
30 cpr.mp.
31 basic life support.mp.
32 life support care.mp.
33 advanced life support.mp.
34 advanced cardiac life support.mp.
35 (bls or als).mp.
36 chest compression*.mp.
37 heart massage.mp. or exp heart massage/
38 or/27-37
39 26 and 38
40 (terminat* or cease or cessation or stop or withdraw* or withhold* or withheld or TOR or futile or futility or rule* or algorithm* or decease* or decision* or prognosis or predict*).mp.
41 exp prognosis/
42 40 or 41
43 39 and 42
44 (letter or editorial).pt.
45 43 not 44
46 limit 45 to human

© 2024 Smyth MA et al. *JAMA Network Open.*

eTable 4. CINAHL Search Strategy

S1	"out of hospital cardiac arrest"
S2	"ohca"
S3	(MH "Ventricular Fibrillation") OR "ventricular fibrillation"
S4	(MH "Tachycardia, Ventricular") OR "ventricular tachycardia"
S5	(MH "Heart Arrest+") OR "heart arrest"
S6	"cardiopulmonary arrest"
S7	"circulatory arrest"
S8	"cardiac standstill"
S9	"pulseless electrical activity"
S10	"pea"
S11	"pulseless"
S12	"shockable"
S13	"non-shockable" or "shockable"
S14	"cardiac arrest"
S15	S3 OR S4 OR S5 OR S6 OR S7 OR S8 OR S9 OR S10 OR S11 OR S12 OR S13 OR S14
S16	"out of hospital" or "out-of-hospital" or "pre-hospital" or "pre hospital" or prehospital
S17	(MH "Emergency Medical Services+") OR "emergency medical service*"
S18	(MH "Emergency Medical Technicians") OR "paramedic*" OR (MH "Prehospital Care")
S19	(MH "Ambulances") OR "ambulance*" or HEMS
S20	"ems or emt"
S21	"field"
S22	S16 OR S17 OR S18 OR S19 OR S20 OR S21
S23	S15 AND S22
S24	S1 OR S2 OR S23
S25	(MH "Resuscitation+") OR (MH "Resuscitation Orders") OR "resuscitat*"
S26	(MH "Resuscitation, Cardiopulmonary+") OR "cardiopulmonary resuscitation"
S27	"cpr"
S28	(MH "Advanced Cardiac Life Support+") OR (MH "Life Support Care+") OR "basic life support"
S29	"advanced life support"
S30	"als or bls"
S31	"chest compression*"
S32	(MH "Heart Massage") OR "heart massage"
S33	S25 OR S26 OR S27 OR S28 OR S29 OR S30 OR S31 OR S32
S34	S24 AND S33
S35	terminat* or cease or cessation or stop or withdraw* or withheld or withhold* or TOR or futile or futility or rule* or algorithm* or decease* or decision* or prognosis or predict*
S36	(MH "Prognosis+")
S37	S35 OR S36
S38	S34 AND S37

© 2024 Smyth MA et al. *JAMA Network Open.*

eTable 5. Cochrane Search Strategy

#1 MeSH descriptor: [Out-of-Hospital Cardiac Arrest] explode all tree
#2 ("OHCA"):ti,ab,kw
#3 MeSH descriptor: [Ventricular Fibrillation] explode all trees
#4 MeSH descriptor: [Tachycardia, Ventricular] explode all trees
#5 (ventricular fibrillation):ti,ab,kw
#6 (ventricular tachycardia):ti,ab,kw
#7 (heart arrest):ti,ab,kw
#8 MeSH descriptor: [Heart Arrest] explode all trees
#9 (cardiopulmonary arrest):ti,ab,kw
#10 (circulatory arrest):ti,ab,kw
#11 ("cardiac standstill"):ti,ab,kw
#12 ("pulseless electrical activity"):ti,ab,kw
#13 (PEA):ti,ab,kw
#14 (pulseless):ti,ab,kw
#15 (shockable):ti,ab,kw
#16 (non-shockable or non shockable):ti,ab,kw
#17 (cardiac arrest):ti,ab,kw
#18 #3 or #4 or #5 or #6 or #7 or #8 or #9 or #10 or #11 or #12 or #13 or #14 or #15 or #16 or #17
#19 (out of hospital or out-of-hospital or pre-hospital or pre hospital or prehospital):ti,ab,kw
#20 MeSH descriptor: [Emergency Medical Services] explode all trees
#21 (emergency medical service*):ti,ab,kw
#22 (paramedic*):ti,ab,kw
#23 MeSH descriptor: [Emergency Medical Technicians] explode all trees
#24 (ambulance*):ti,ab,kw
#25 MeSH descriptor: [Ambulances] explode all trees
#26 (ems or emt):ti,ab,kw
#27 field
#28 #19 or #20 or #21 or #22 or #23 or #24 or #25 or #26 or #27
#29 #18 and #28
#30 #1 or #2 or #29
#31 (resuscitat*):ti,ab,kw
#32 MeSH descriptor: [Resuscitation] explode all trees
#33 MeSH descriptor: [Resuscitation Orders] explode all trees
#34 MeSH descriptor: [Cardiopulmonary Resuscitation] explode all trees
#35 (cardiopulmonary resuscitation):ti,ab,kw
#36 ("CPR"):ti,ab,kw
#37 (basic life support):ti,ab,kw
#38 MeSH descriptor: [Life Support Care] explode all trees
#39 (advanced life support):ti,ab,kw
#40 MeSH descriptor: [Advanced Cardiac Life Support] explode all trees
#41 (als or bls):ti,ab,kw
#42 MeSH descriptor: [Heart Massage] explode all trees
#43 (chest compression*):ti,ab,kw
#44 #31 or #32 or #33 or #34 or #35 or #36 or #37 or #38 or #39 or #40 or #41 or #42 or #43
#45 #30 and #44
#46 (terminat* or cease or cessation or stop or withdraw* or withhold* or withheld or TOR or futile or futility or rule* or algorithm* or decease* or decision* or prognosis or predict*):ti,ab,kw
#47 MeSH descriptor: [Prognosis] explode all trees
#48 #46 or #47
#49 #45 and #48

© 2024 Smyth MA et al. *JAMA Network Open.*

eTable 6. Web of Science Search Strategy

#7 #6 AND #5
Indexes=SCI-EXPANDED, SSCI, CPCI-S, CPCI-SSH Timespan=1900-2023
#6 TOPIC: (terminat* or cease or cessation or stop or withdraw* or withheld or withhold* or TOR or futile or futility or rule* or algorithm* or decease* or decision* or prognosis or predict*)
Indexes=SCI-EXPANDED, SSCI, CPCI-S, CPCI-SSH Timespan=1900-2023
#5 #4 AND #3
Indexes=SCI-EXPANDED, SSCI, CPCI-S, CPCI-SSH Timespan=1900-2023
#4 TOPIC: (resuscitat* or cpr or "basic life support" or "life support care" or "advanced cardiac life support" or "advanced life support" or bls or als or "heart massage" or "chest compression*")
Indexes=SCI-EXPANDED, SSCI, CPCI-S, CPCI-SSH Timespan=1900-2023
#3 #2 OR #1
Indexes=SCI-EXPANDED, SSCI, CPCI-S, CPCI-SSH Timespan=1900-2023
#2 TS=("ventricular fibrillation" or "ventricular tachycardia" or "heart arrest" or "cardiac arrest" or "cardiopulmonary arrest" or "circulatory arrest" or "cardiac standstill" or "pulseless electrical activity" or pea or pulseless or shockable or non-shockable or nonshockable) AND TS=("out of hospital" or "out-of-hospital" or prehospital or "pre-hospital" or "pre hospital" or "emergency medical service*" or "emergency medical technician*" or paramedic* or ambulance* or ems or emt or field or HEMS)
Indexes=SCI-EXPANDED, SSCI, CPCI-S, CPCI-SSH Timespan=1900-2023
#1 TOPIC: ("out of hospital cardiac arrest" or "out-of-hospital cardiac arrest" or ohca)
Indexes=SCI-EXPANDED, SSCI, CPCI-S, CPCI-SSH Timespan=1900-2023

© 2024 Smyth MA et al. *JAMA Network Open.*

eTable 7. Derivation Studies Paired Sensitivity and Specificity

Author (year)	TOR Rule	Prevalence	Population	TP	FP	FN	TN	Sensitivity [95% CI]	Specificity [95% CI]
Morrison et al,[1] (2007)	ALS	94.9%	Adult (cardiac)	1425	0	3009	239	0.32 [0.31-0.34]	1.00 [0.98-1.00]
Verbeek et al,[2] (2002)	BLS	98.0%	Adult (medical)	425	0	224	13	0.65 [0.62-0.69]	0.99 [0.74-1.00]
Glober et al,[3] (2019)	Glober 1	90.9%	Adult (medical & trauma)	223	0	1327	156	0.14 [0.13-0.16]	1.00 [0.98-1.00]
Goto et al,[4] (2019)	Goto 1	95.8%	Adult (EMS witnessed)	11069	21	90746	4434	0.11 [0.11-0.11]	1.00 [0.99-1.00]
Haukoos et al,[5] (2004)	Haukoos 1	95.2%	Adult (medical)	487	3	231	33	0.68 [0.64-0.71]	0.92 [0.78-0.98]
Jabre et al,[6] (2016)	Jabre	89.5%	Adult (cardiac)	2799	1	3435	728	0.45 [0.44-0.46]	1.00 [0.99-1.00]
Yoon et al,[7] (2019)	KoCARC 1	88.5%	Adult (cardiac)	2037	40	1820	463	0.53 [0.51-0.54]	0.92 [0.89-0.94]
Yoon et al,[7] (2019)	KoCARC 2	88.5%	Adult (cardiac)	2027	56	1830	447	0.53 [0.51-0.54]	0.89 [0.86-0.91]
Yoon et al,[7] (2019)	KoCARC 3	88.5%	Adult (cardiac)	1512	25	2345	478	0.39 [0.38-0.41]	0.95 [0.93-0.97]
Lee et al,[8] (2019)	KoCARC 4	88.3%	Adult (cardiac)	1245	14	2825	524	0.31 [0.29-0.32]	0.97 [0.96-0.99]
Lee et al,[8] (2019)	KoCARC 5	88.3%	Adult (cardiac)	1316	11	2754	527	0.32 [0.31-0.34]	0.98 [0.96-0.99]
Marsden et al,[9] (1995)	Marsden	99.7%	Adult (medical & trauma)	240	0	173	1	0.58 [0.53-0.63]	0.91 [0.02-1.00]
House et al,[10] (2018)	PEA	84.3%	Adult (cardiac, transported)	829	3	955	328	0.46 [0.44-0.49]	0.99 [0.97-1.00]
Petrie et al,[11] (2001)	Petrie	95.7%	Adult (cardiac)	3720	7	5751	421	0.39 [0.38-0.40]	0.98 [0.97-0.99]
SOS-Kanto,[12] (2017)	SOS Kanto 1	95.2%	Adult (medical & trauma)	6274	34	6385	598	0.50 [0.49-0.50]	0.95 [0.93-0.96]
SOS-Kanto,[12] (2017)	SOS Kanto 2	95.2%	Adult (medical & trauma)	5609	17	7050	615	0.44 [0.43-0.45]	0.97 [0.96-0.98]
SOS-Kanto,[12] (2017)	SOS Kanto 3	95.2%	Adult (medical & trauma)	5196	9	7463	623	0.41 [0.40-0.42]	0.99 [0.97-0.99]
Shibahashi et al,[13] (2020)	Shibahashi 3	96.6%	Child (trauma)	422	5	140	15	0.75 [0.71-0.79]	0.75 [0.51-0.91]
Bonnin et al,[14] (1993)	no ROSC	93.0%	Adult (medical)	946	6	284	86	0.77 [0.74-0.79]	0.93 [0.86-0.98]
Chiang et al,[15] (2016)	tCPA	95.6%	Adult (trauma)	147	0	707	39	0.17 [0.15-0.20]	1.00 [0.91-1.00]

True positive (TP) means TOR rule indicates stop and patient dies, False positive (FP) TOR rule indicates stop but patient survives (missed survivors) True negative (TN) TOR rule indicates continue and patient survives, False negative (FN) TOR rule indicates continue but patient dies (futile resuscitations) Sensitivity is proportion of cases where the TOR indicates stop out of all deaths. Specificity is proportion of cases where the TOR rule indicates continue out of all survivors.

eTable 8. External Validation Studies Paired Sensitivity and Specificity

Author (year)	TOR Rule	Prevalence	Population	TP	FP	FN	TN	Sensitivity [95% CI]	Specificity [95% CI]
Cheong et al,[16] (2016)	ALS	96.5%	Adult (cardiac)	586	1	1531	75	0.28 [0.26-0.30]	0.99 [0.93-1.00]
Chiang et al,[17] (2015)	ALS	94.4%	Adult (medical)	1896	48	1396	149	0.58 [0.56-0.59]	0.76 [0.69-0.81]
Diskin et al,[18] (2014)	ALS	87.9%	Adult (cardiac)	75	0	208	39	0.27 [0.21-0.32]	1.00 [0.91-1.00]
Fukada et al,[19] (2014)	ALS	90.0%	Adult (medical & trauma)	7	0	30	4	0.19 [0.08-0.35]	0.98 [0.38-1.00]
House et al,[10] (2018)	ALS	84.4%	Adult (cardiac, transported)	102	0	1695	331	0.06 [0.05-0.07]	1.00 [0.99-1.00]
Hreinsson et al,[20] (2020)	ALS	79.9%	Adult (cardiac)	35	0	414	113	0.08 [0.05-0.11]	1.00 [0.97-1.00]
Hsu et al,[21] (2022)	ALS	94.0%	Adult (medical)	25164	385	26613	2902	0.49 [0.48-0.49]	0.88 [0.87-0.89]
Kajino et al,[22] (2013)	ALS	96.0%	Adult (cardiac)	40612	418	91919	5037	0.31 [0.30-0.31]	0.92 [0.92-0.93]
Kashiura et al,[23] (2016)	ALS	96.2%	Adult (medical)	1681	20	4170	210	0.29 [0.28-0.30]	0.91 [0.87-0.95]
Lee et al,[8] (2019)	ALS	88.3%	Adult (cardiac)	868	17	3202	521	0.21 [0.20-0.23]	0.97 [0.95-0.98]
Lin et al,[24] (2022)	ALS	93.6%	Adult (2015 cohort)	122	2	231	22	0.35 [0.30-0.40]	0.92 [0.73-0.99]
Lin et al,[24] (2022)	ALS	94.1%	Adult (2020 cohort)	104	0	279	24	0.27 [0.23-0.32]	1.00 [0.85-1.00]
Matsui et al,[25] (2023)	ALS	88.5%	Child (medical & trauma)	299	21	1319	190	0.18 [0.17-0.20]	0.90 [0.85-0.94]
Morrison et al,[26] (2009)	ALS	94.6%	Adult (cardiac)	743	0	1523	130	0.33 [0.31-0.35]	1.00 [0.97-1.00]
Sasson et al,[27] (2008)	ALS	92.9%	Adult (cardiac)	1192	0	3921	392	0.23 [0.22-0.24]	1.00 [0.99-1.00]
Skrifvars et al,[28] (2010)	ALS	98.9%	Adult (medical, non-shockable)	5464	2	15020	219	0.27 [0.26-0.27]	0.99 [0.97-1.00]
Smits et al,[29] (2023)	ALS	87.5%	Adult (cardiac, male)	3834	6	15240	2728	0.20 [0.20-0.21]	1.00 [1.00-1.00]
Smits et al,[29] (2023)	ALS	92.9%	Adult (cardiac, female)	2301	3	7704	764	0.23 [0.22-0.24]	1.00 [0.99-1.00]
Verhaert et al,[30] (2016)	ALS	84.8%	Adult (medical)	35	0	464	89	0.07 [0.05-0.10]	1.00 [0.96-1.00]
Chiang et al,[17] (2015)	BLS	94.4%	Adult (medical)	2105	51	1187	146	0.64 [0.62-0.66]	0.74 [0.67-0.80]
Kim et al,[31] (2015)	BLS	90.7%	Adult (cardiac)	3224	137	1160	314	0.74 [0.72-0.75]	0.70 [0.65-0.74]
Lee et al,[8] (2019)	BLS	88.3%	Adult (cardiac)	2921	118	1149	420	0.72 [0.70-0.73]	0.78 [0.74-0.81]
Matsui et al,[25] (2023)	BLS	85.3%	Child (medical & trauma)	5474	440	869	657	0.86 [0.85-0.87]	0.60 [0.57-0.63]
Ong et al,[32] (2006)	BLS	95.4%	Adult (cardiac)	6905	3	6143	633	0.53 [0.52-0.54]	1.00 [0.99-1.00]
Ong et al,[33] (2007)	BLS	98.6%	Adult (medical)	1553	6	684	26	0.69 [0.67-0.71]	0.81 [0.64-0.93]
Skrifvars et al,[28] (2010)	ERC	98.9%	Adult (medical, non-shockable)	19354	11	1130	210	0.94 [0.94-0.95]	0.95 [0.91-0.97]
Glober et al,[34] (2020)	Glober 1	91.5%	Adult (medical & trauma)	290	0	3407	344	0.08 [0.07-0.09]	1.00 [0.99-1.00]
Hsu et al,[21] (2022)	Goto 1	94.0%	Adult (medical)	27856	283	23921	3004	0.54 [0.53-0.54]	0.91 [0.90-0.92]
Lee et al,[8] (2019)	Goto 1	88.3%	Adult (cardiac)	1579	27	2491	511	0.39 [0.37-0.40]	0.95 [0.93-0.97]

© 2024 Smyth MA et al. *JAMA Network Open.*

eTable 8. External Validation Studies Paired Sensitivity and Specificity

Author (year)	TOR Rule	Prevalence	Population	TP	FP	FN	TN	Sensitivity [95% CI]	Specificity [95% CI]
SOS-Kanto,[12] (2017)	Goto 2	95.2%	Adult (medical & trauma)	6319	33	6340	599	0.50 [0.49-0.51]	0.95 [0.93-0.96]
Skrifvars et al,[28] (2010)	Helsinki	98.9%	Adult (medical, non-shockable)	11258	57	9226	164	0.55 [0.54-0.56]	0.74 [0.68-0.80]
Hreinsson et al,[20] (2020)	Jabre	80.1%	Adult (cardiac)	215	0	240	113	0.47 [0.43-0.52]	1.00 [0.97-1.00]
Park et al,[35] (2023)	KoCARC 1	93.4%	Adult (medical)	668	7	1039	113	0.39 [0.37-0.41]	0.94 [0.88-0.98]
Park et al,[35] (2023)	KoCARC 2	93.4%	Adult (medical)	687	11	1020	109	0.40 [0.38-0.43]	0.91 [0.84-0.95]
Park et al,[35] (2023)	KoCARC 3	93.4%	Adult (medical)	524	6	1183	114	0.31 [0.29-0.33]	0.95 [0.89-0.98]
Harris et al,[36] (2021)	MIEMS	71.0%	Child (trauma, age 0-17)	27	4	71	36	0.28 [0.19-0.37]	0.90 [0.76-0.97]
Harris et al,[36] (2021)	MIEMS	73.0%	Child (trauma, age 0-14)	39	4	107	50	0.27 [0.20-0.35]	0.93 [0.82-0.98]
Harris (2021)	MIEMS	76.8%	Child (medical, age 0-17)	44	1	1028	322	0.04 [0.03-0.05]	1.00 [0.98-1.00]
Ong et al,[32] (2006)	Marsden	95.4%	Adult (cardiac)	2535	1	10513	635	0.19 [0.19-0.20]	1.00 [0.99-1.00]
Ong et al,[33] (2007)	Marsden	98.6%	Adult (medical)	1447	3	790	29	0.65 [0.63-0.67]	0.91 [0.75-0.98]
Cone et al,[37] (2005)	NAEMSP	97.6%	Adult (medical)	285	0	204	12	0.58 [0.54-0.63]	0.99 [0.72-1.00]
Ong et al,[32] (2006)	Petrie	95.4%	Adult (cardiac)	1292	1	11756	635	0.10 [0.09-0.10]	1.00 [0.99-1.00]
Ong et al,[33] (2007)	Petrie	98.6%	Adult (medical)	716	2	1521	30	0.32 [0.30-0.34]	0.94 [0.79-0.99]
Lee et al,[8] (2019)	SOS Kanto 1	88.3%	Adult (cardiac)	1102	9	2968	529	0.27 [0.26-0.28]	0.98 [0.97-0.99]
Grunau et al,[38] (2019)	Shibahashi 1	90.7%	Adult (medical)	5378	64	18090	2343	0.23 [0.22-0.23]	0.97 [0.97-0.98]
Hsu et al,[21] (2022)	Shibahashi 1	94.0%	Adult (medical)	18122	191	33655	3096	0.35 [0.35-0.35]	0.94 [0.93-0.95]
Cheong et al,[16] (2016)	uTOR	96.5%	Adult (cardiac)	1406	5	711	71	0.66 [0.64-0.68]	0.93 [0.85-0.98]
Drennan et al,[39] (2014)	uTOR	97.7%	Adult (cardiac)	2471	15	3234	122	0.43 [0.42-0.45]	0.89 [0.83-0.94]
Fukada et al,[19] (2014)	uTOR	95.9%	Adult (medical & trauma)	100	1	42	5	0.70 [0.62-0.78]	0.83 [0.36-1.00]
Goto et al,[4] (2019)	uTOR	95.8%	Adult (EMS witnessed)	92581	1714	9234	2741	0.91 [0.91-0.91]	0.62 [0.60-0.63]
Grunau et al,[40] (2017)	uTOR	85.1%	Adult (medical)	4275	92	1674	953	0.72 [0.71-0.73]	0.91 [0.89-0.93]
House et al,[10] (2018)	uTOR	84.5%	Adult (cardiac, transported)	582	0	1226	331	0.32 [0.30-0.34]	1.00 [0.99-1.00]
Hreinsson et al,[20] (2020)	uTOR	80.1%	Adult (cardiac)	202	0	252	113	0.44 [0.40-0.49]	1.00 [0.97-1.00]
Hsu et al,[21] (2022)	uTOR	94.0%	Adult (medical)	40904	657	10873	2630	0.79 [0.79-0.79]	0.80 [0.79-0.81]
Jordan et al,[41] (2017)	uTOR	81.9%	Adult (medical)	22	0	69	20	0.24 [0.16-0.34]	1.00 [0.82-1.00]
Kajino et al,[22] (2013)	uTOR	93.7%	Adult (cardiac)	111980	1160	29656	8356	0.79 [0.79-0.79]	0.88 [0.87-0.88]
Kashiura et al,[23] (2016)	uTOR	93.7%	Adult (medical)	4165	28	917	314	0.82 [0.81-0.83]	0.92 [0.88-0.94]
Lin et al,[24] (2022)	uTOR	96.4%	Adult (2015 cohort)	738	19	113	13	0.87 [0.84-0.89]	0.41 [0.24-0.59]
Lin et al,[24] (2022)	uTOR	95.5%	Adult (2020 cohort)	430	8	116	18	0.79 [0.75-0.82]	0.69 [0.48-0.86]

© 2024 Smyth MA et al. *JAMA Network Open.*

eTable 8. External Validation Studies Paired Sensitivity and Specificity

Author (year)	TOR Rule	Prevalence	Population	TP	FP	FN	TN	Sensitivity [95% CI]	Specificity [95% CI]
Morrison et al,[26] (2009)	uTOR	94.6%	Adult (cardiac)	1302	0	964	130	0.57 [0.55-0.60]	1.00 [0.97-1.00]
Morrison et al,[1] (2007)	uTOR	94.9%	Adult (cardiac)	2263	0	2171	239	0.51 [0.50-0.53]	1.00 [0.98-1.00]
Park et al,[35] (2023)	uTOR	93.4%	Adult (medical)	877	14	830	106	0.51 [0.49-0.54]	0.88 [0.81-0.93]
Sasson et al,[27] (2008)	uTOR	92.9%	Adult (cardiac)	2587	5	2526	387	0.51 [0.49-0.52]	0.99 [0.97-1.00]
SOS-Kanto,[12] (2017)	uTOR	95.2%	Adult (medical & trauma)	9892	72	2767	560	0.78 [0.77-0.79]	0.89 [0.86-0.91]
Yates et al,[42] (2018)	uTOR	98.6%	Adult (transported)	83	0	134	3	0.38 [0.32-0.45]	0.97 [0.28-1.00]
Yoon et al,[7] (2019)	uTOR	88.5%	Adult (cardiac)	2704	97	1153	406	0.70 [0.69-0.72]	0.81 [0.77-0.84]
Hreinsson et al,[20] (2020)	uTOR OR Jabre	80.1%	Adult (cardiac)	270	0	184	113	0.59 [0.55-0.64]	1.00 [0.97-1.00]

True positive (TP) means TOR rule indicates stop and patient dies, False positive (FP) TOR rule indicates stop but patient survives (missed survivors) True negative (TN) TOR rule indicates continue and patient survives, False negative (FN) TOR rule indicates continue but patient dies (futile resuscitations) Sensitivity is proportion of cases where the TOR indicates stop out of all deaths. Specificity is proportion of cases where the TOR rule indicates continue out of all survivors.

eTable 9. Clinical Studies Paired Sensitivity and Specificity

Author (year)	TOR Rule	Prevalence	Population	TP	FP	FN	TN	Sensitivity [95% CI]	Specificity [95% CI]
Morrison et al,[43] (2014)	BLS	95.4%	Adult (medical)	586	0	323	44	0.64 [0.61-0.68]	1.00 [0.92-1.00]

True positive (TP) means TOR rule indicates stop and patient dies, False positive (FP) TOR rule indicates stop but patient survives (missed survivors) True negative (TN) TOR rule indicates continue and patient survives, False negative (FN) TOR rule indicates continue but patient dies (futile resuscitations) Sensitivity is proportion of cases where the TOR indicates stop out of all deaths. Specificity is proportion of cases where the TOR rule indicates continue out of all survivors.

© 2024 Smyth MA et al. *JAMA Network Open.*

eTable 10. Summary of Included Studies

Author (year), country	Study type<br>TOR rule (n)	Population	EMS system
Bonnin et al,[14] (1993), USA	Derivation study<br>No ROSC TOR (n=1322)	Retrospective analysis of city of Houston cardiac arrest registry. Excluded patient groups:<br>• Obviously deceased<br>• Age < 18<br>• Persistent VF<br>• Trauma aetiology<br>• Hypothermia<br>• Primary respiratory aetiology<br>• Overdose	Tiered EMS system. BLS first response with paramedic ALS secondary response
Cheong et al,[16] (2016), Singapore	Validation study<br>BLS TOR (n=2193)**<br>ALS TOR (n=2193)	Retrospective analysis of all OHCA patients transported by EMS to 7 EDs participating in the PAROS study. Excluded patient groups:<br>• Obviously deceased<br>• Age < 18<br>• DNACPR<br>• Non-cardiac aetiology	Single tier EMS system. EMTs provide intermediate life support including AED, LMA, IV adrenaline. No manual defibrillation, no ETI, no amiodarone/lignocaine
Chiang et al,[17] (2015), Taiwan	Validation study<br>BLS TOR (n=3489)**<br>ALS TOR (n=3489)	Retrospective analysis of city of Taipei Fire Department cardiac arrest registry. Excluded patient groups:<br>• Obviously deceased<br>• DNACPR<br>• Age < 18<br>• Trauma aetiology	Mixed EMS response may include BLS only, ALS only or mixed BLS/ALS. BLS level providers can provide defibrillation, while ALS providers can perform ETI and administer ACLS medications
Chiang et al,[15] (2016), Taiwan	Derivation study<br>tCPA TOR (n=893)	Retrospective analysis of city of Taipei Fire Department cardiac arrest registry. Excluded patient groups:<br>• Obviously deceased<br>• DNACPR<br>• Age < 18<br>• Non-trauma aetiology	Not reported. However, a prior study[xx] by the same author, using the same registry, indicates a mixed EMS response that may include BLS only, ALS only or mixed BLS/ALS. BLS level providers can provide defibrillation, while ALS providers

© 2024 Smyth MA et al. *JAMA Network Open.*

		Note – FBAO and intoxication classified as non-blunt injury and conflated as penetrating trauma	can perform ETI and administer ACLS medications
Cone et al,[37] (2005), USA	Validation study<br>NAEMSP TOR (n=501)	Both prospective and retrospective data utilised. Retrospective data from a single hospital database. Prospective data collected at two hospitals (one of which provided the retrospective data). Excluded patient groups:<br>• DNACPR<br>• Age < 18<br>• Hypothermia<br>• Cold- water drowning	Fire base ALS first response with BLS or ALS EMS transport
Diskin et al,[18] (2014), USA	Validation study<br>ALS TOR (n=322)	Retrospective analysis of Richmond EMS cardiac arrest records. Excluded patient groups:<br>• Obviously deceased<br>• DNACPR<br>• Age <18<br>• Non-cardiac aetiology<br>• Trauma aetiology<br>• Pregnant	Single tier ALS EMS system. Intra arrest management included an aggressive vasopressor regimen combining both adrenaline and vasopressin, cold saline infusion and mechanical CPR (Autopulse).
Drennan et al,[39] (2014), Canada	Validation study<br>uTOR (n=5842)	Retrospective analysis of the Toronto Resuscitation Outcomes Consortium (ROC) Epistry database. Only patients transported (not terminated on scene due to futility) were included. Excluded patient groups:<br>• Obviously deceased<br>• DNACPR<br>• Terminated on scene (futile)<br>• Age <18<br>• Non-cardiac aetiology<br>• Trauma aetiology<br>• Drowning<br>• Asphyxia<br>• Overdose	Mixed BLS and ALS EMS system with existing termination of resuscitation protocol. Nearest ambulance assigned; all cardiac arrest cases assigned an ALS ambulance if BLS resource is closest. BLS level providers can provide semi-automatic defibrillation and King airway, while ALS providers can perform manual defibrillation, ETI, IV/IO access and administer ACLS medications
Fukada et al,[19] (2014), Japan	Validation study<br>BLS TOR (n=148)**<br>ALS TOR (n=41)	Retrospective analysis of cases transported to University of Tokyo Hospital. Excluded patient groups:	Single tier EMS system. EMTs can provide defibrillation, ETI, IV access, IV infusion, IV adrenaline

© 2024 Smyth MA et al. *JAMA Network Open.*

		• Age < 18	
Glober et al,[3] (2019), USA	Derivation study<br>Glob 1 TOR (n=1706)	Retrospective analysis of cases attended by San Mateo County EMS using data reported to the CARES registry. No excluded patient groups reported.	ALS EMS system
Glober et al,[34] (2020), USA	Validation study<br>Glob 1 TOR (n=4041)	Retrospective analysis of cases in Marion County, Indiana attended by Indianapolis EMS using routinely collected data. Excluded patient groups:<br>• Age < 18	ALS EMS system
Goto et al,[4] (2019), Japan	Derivation study<br>Goto 1 TOR (RIP, n=106270)<br>Validation study<br>BLS (n=106270)**	Retrospective analysis of National FDMA database. Excluded patient groups:<br>• EMS witnessed cardiac arrest<br>• Age < 18	Single tier EMS system. EMT level providers can provide defibrillation, airway adjunct insertion, IV access, IV infusion. ALS providers can perform ETI and administer adrenaline. EMS personnel cannot legally terminate resuscitation
Grunau et al,[40] (2017), Canada	Validation study<br>uTOR (n=6994)	Prospectively collected data in four metropolitan regions of British Columbia. Excluded patient groups:<br>• DNACPR<br>• Age <18<br>• Trauma aetiology	Tiered response. Fire first responders supported by EMS ALS secondary response. First reponders can provide defibrillation using an AED. ALS responders can provide manual defib, ETI, cricothyroidotomy, IV & IO access, IV fluids, ACLS medications

© 2024 Smyth MA et al. *JAMA Network Open.*

Grunau et al,[38] (2019), Canada	Validation study<br>Shibahashi 1 TOR<br>(n=25875)	Secondary analysis of patients recruited to the Resuscitation Outcomes Consortium continuous versus interrupted chest compressions (CCC) trial. Excluded patient groups:<br>• EMS-witnessed arrest<br>• DNACPR<br>• Trauma Aetiology<br>• Uncontrolled bleeding or exsanguination<br>• Asphyxia<br>• Pregnant<br>• Pre-existing tracheostomy<br>• Prisoners<br>• Mechanical chest compression device<br>• Advanced airway management before EMS arrival<br>• Opted out of resuscitation research	ALS EMS system
Harris et al,[36] (2021), USA	Validation study<br>MIEMS Medical (<18)<br>(n=1,395)<br>MIEMS Trauma (<18)<br>(n=200)<br>MIEMS Trauma (<15)<br>(n=138)	Retrospective analysis of cases within Electronic Health Records. Excluded patient groups:<br>• Adult<br>• Interfacility transports	ALS EMS system
Haukoos et al,[5] (2004), USA	Derivation study<br>Haukoos 1 TOR (n=754)	Retrospective analysis of cases transported to a single hospital. Excluded patient groups:<br>• Age <18<br>• Trauma aetiology	ALS EMS system
House et al,[10] (2018), UK	Derivation study<br>PEA TOR (n=2,115)<br>Validation study<br>BLS (n=2,139)**<br>ALS TOR (n=2,128)	Retrospective analysis of cases transported by a single EMS.	ALS EMS system
	Validation study		ALS EMS system

© 2024 Smyth MA et al. *JAMA Network Open.*

Hreinsson et al,[20] (2020), Iceland	BLS (n=568)**<br>ALS TOR (n=568)<br>Jabre TOR (n=568)<br>BLS or Jabre TOR (n=568) **	Retrospective analysis of VAKI database.<br>Excluded patient groups:<br>• Age < 18<br>• Obviously deceased<br>• DNACPR<br>• Non-cardiac aetiology<br>• Outside Reykjavik metropolitan area	
Hsu et al,[21] (2022), Taiwan	Validation study<br>BLS (n=55,064)**<br>ALS (n=55,064)<br>Goto 2 (n=55,064)<br>Shibahashi 1 (n=55,064)	Retrospective analysis of PAROS database.<br>Excluded patient groups:<br>• Age < 18<br>• Non-EMS transported<br>• Obviously deceased<br>• DNACPR<br>• Trauma<br>• Missing data	
Jabre et al,[6] (2016), France	Derivation study<br>Jabre (n=6,963)	Retrospective analysis<br>• Age <18<br>• Non-cardiac cause<br>• Terminal illness<br>• DNACPR	Analysis of data from PRESENCE study and King County data. In Paris EMS response included physicians. In the USA there was a tiered ALS response.
Jordan et al,[41] (2017), USA	Validation study<br>uTOR (n=111)	Retrospective analysis of cases transported to a single hospital. Excluded patient groups:<br>• Obviously deceased<br>• DNACPR<br>• Trauma aetiology<br>• Hanging<br>• Drowning	14 EMS agencies with a mix of response models. Participating clinicians include 35 Paramedics, 256 AEMTs, 240 EMTs, and 27 Emergency Medical Responders.
Kajino et al,[22] (2013), Japan	Validation study<br>BLS (n=151152)**<br>ALS TOR (n=137986)	Retrospective analysis of National FDMA database. Excluded patient groups:<br>• Age < 18<br>• Non-cardiac aetiology<br>• Cerebrovascular aetiology<br>• Respiratory aetiology<br>• Asphyxia<br>• Hanging<br>• Malignant tumour	Single tier EMS system. EMT level providers can provide defibrillation, airway adjunct insertion, IV access, IV infusion. ALS providers can perform ETI and administer adrenaline. EMS personnel cannot legally terminate resuscitation

© 2024 Smyth MA et al. *JAMA Network Open.*

		• Trauma aetiology	
Kashiura et al,[23] (2016), Japan	Validation study<br>BLS TOR (n=5,424) **<br>ALS TOR[b] (n=6,081)	Retrospective analysis of National FDMA database. Excluded patient groups:<br>• Age < 18<br>• Trauma aetiology	Single tier EMS system. EMT level providers can provide defibrillation, airway adjunct insertion, IV access, IV infusion. ALS providers can perform ETI and administer adrenaline. EMS personnel cannot legally terminate resuscitation
Kim et al,[31] (2015), Korea	Validation study<br>BLS TOR (n=4,835)	Retrospective analysis of cases attended by Seoul Metropolitan Fire Department (SMFD). Excluded patient groups:<br>• Age < 19<br>• Non-cardiac aetiology	Single tier BLS EMS system
Lee et al,[8] (2019), Korea	Derivation study<br>KoCARC 4 TOR (n=4,608)<br>KoCARC 5 TOR (n=4,608)<br>Validation study<br>BLS TOR (n=4,608)<br>ALS TOR (n=4,608)<br>Goto 1 TOR (n=4,608)<br>SOS-Kanto 1 TOR (n=4,608)	Retrospective analysis of Korean nationwide registry. Excluded patient groups:<br>• Obviously deceased<br>• Age < 18<br>• Terminal illness/hospice care/DNAR<br>• Non-cardiac aetiology<br>• Pregnant<br>• Trauma aetiology<br>• Electrocution<br>• Hypothermia<br>• Primary respiratory aetiology<br>• Drowning<br>• Asphyxia<br>• Poisoning	Not defined. EMS personnel cannot legally terminate resuscitation and can use a manual defibrillator.
Lin et al,[24] (2022), Taiwan	Validation study<br>BLS (n=883) (2015 cohort)<br>BLS (n=572) (2020 cohort)<br>ALS (n=377) (2015 cohort)<br>ALS (n=407) (2020 cohort)	Restrospective analysis. Excluded patient groups:<br>• Age < 18<br>• Obviously deceased<br>• DNACPR<br>• Trauma	Two tiered EMS system. BLS rule applied by BLS crews, ALS rule applied by ALS crews

© 2024 Smyth MA et al. *JAMA Network Open.*

Marsden et al,[9] (1995), UK	Derivation study<br>Marsden TOR (n=414)	Retrospective analysis of HeartStart Scotland database. Excluded patient groups:<br>• Obviously deceased<br>• Age < 18<br>• Pregnant<br>• Poisoning or overdose<br>• Hypothermia<br>• Drowning	Single tier ALS EMS system
Matsui et al,[25] (2023)	Validation study<br>BLS TOR (n=7,440)<br>ALS TOR (n=1,829)	Retrospective analysis of FDMA registry. Excluded patient groups:<br>• Age > 17<br>• Obviously deceased	ALS capable EMS system. The BLS rule was applied to patients who received BLS level care only (irrespective of provider training). The ALS rule was applied to patients who received ALS level care
Morrison et al,[1] (2007), Canada	Validation study<br>BLS TOR (n=4,673)<br>ALS TOR (n=4,673)	Retrospective analysis of OPALS study database (phase III - ALS). Excluded patient groups:<br>• Non-cardiac aetiology (including trauma, drowning, poisoning, hypothermia)<br>• Obviously deceased (rigor mortis, lividity, decomposition, decapitation)<br>• Age < 16<br>• DNACPR	ALS system
Morrison et al,[26] (2009), Canada	Validation study<br>ALS TOR (n=2,396)<br>uTOR (n=2,396)	Retrospective analysis of the Toronto Resuscitation Outcomes Consortium (ROC) Epistry database. Excluded patient groups:<br>• Non-cardiac aetiology (including trauma, drowning, poisoning, hypothermia)<br>• Obviously deceased (rigor mortis, lividity, decomposition, decapitation)<br>• Age < 16<br>• DNACPR	Tiered response involving police, fire and EMTs with AEDs. Some regions also had ALS paramedics as part of the response.
	Clinical study		

© 2024 Smyth MA et al. *JAMA Network Open.*

Morrison et al,[43] (2014), Canada	BLS TOR (n=953)	Prospective analysis of consecutive cardiac arrest patients within the TORIT trial in seven regions of Ontario. Excluded patient groups:<br>• Received ACLS<br>• Obviously deceased (rigor mortis, lividity, decomposition, decapitation)<br>• Arrest from an obvious cause (e.g. hanging, drowning, trauma)<br>• Age < 16<br>• DNACPR	Mixed EMS system. Only BLS level cases included. Decision to stop after consultation with base physician that occurred following 4x2 minute cycles.
Ong et al,[32] (2006), Canada	Validation study<br>BLS TOR (n=13,684)<br>Marsden TOR (n=13,684)<br>Petrie TOR (n=13,684)	Retrospective analysis of OPALS study database (phase II & III – BLS cases only). Excluded patient groups:<br>• Obviously deceased (rigor mortis, lividity, decomposition, decapitation)<br>• Age < 16<br>• DNACPR<br>• Non-cardiac aetiology (including trauma, drowning, poisoning, hypothermia)<br>• Received ALS on scene	Tiered EMS system. Only BLS level cases included.
Ong et al,[33] (2007), Singapore	Validation study<br>BLS TOR (n=2,269)<br>Marsden TOR (n=2,269)<br>Petrie TOR (n=2,269)	Retrospective analysis of CARE database. Excluded patient groups:<br>• Obviously deceased (rigor mortis, lividity, decomposition, decapitation)	Single tier EMS system. EMTs can provide defibrillation using AEDs.
Park et al,[35] (2023), Japan	Validation study<br>uTOR (n=1,827)<br>KOCARC 1 (n=1,827)<br>KOCARC 2 (n=1,827)<br>KOCARC 3 (n=1,827)	Retrospective analysis. Excluded patient groups:<br>• Age < 18<br>• Trauma, intoxication, or drowning,<br>• Obvioulsy deceased<br>• DNACPR	ALS capable EMS system. Majority of crews were BLS or ILS capable. Few ALS crews.

© 2024 Smyth MA et al. *JAMA Network Open.*

Petrie et al,[11] (2001), Canada	Derivation study<br>Petrie TOR (n=9,899)	Retrospective analysis of OPALS study database (phase I & II – BLS only). Excluded patient groups:<br>• Obviously deceased (rigor mortis, lividity, decomposition, decapitation)<br>• Age < 16<br>• DNACPR<br>• Non-cardiac aetiology (including trauma, drowning, poisoning, hypothermia)<br>• Received ALS on scene	BLS EMS system.
Sasson et al,[27] (2008), USA	Validation study<br>BLS (n=5,505)**<br>ALS TOR (n=5,505)	Retrospective analysis of CARES database. Excluded patient groups:<br>• Obviously deceased (rigor mortis, lividity, decomposition, decapitation)<br>• Age < 16<br>• Non-cardiac aetiology	8 US cities submit data to the CARES registry. There is a mix of EMS response models. Although not explicitly stated it is assumed all cities provide ALS level paramedic response. There are no details addressing single or tiered response systems.
Shibahashi et al,[13] (2020), Japan	Derivation study<br>Shibahashi 3 (n=582)	Retrospective analysis of FDMA database. Excluded patient groups;<br>• Age >17<br>• Non-RTC trauma<br>• Medical	Single tier EMS system. EMT level providers can provide defibrillation, airway adjunct insertion, IV access, IV infusion. ALS providers can perform ETI and administer adrenaline. EMS personnel cannot legally terminate resuscitation
Skrifvars et al,[28] (2010), Sweden	Validation study<br>ALS TOR (n=20,705)<br>ERC TOR (n=20,705)<br>Helsinki TOR (n=20,705)	Retrospective analysis of the Swedish Cardiac Arrest Registry. Excluded patient groups:<br>• EMS witnessed<br>• Presenting in VF or VT<br>• Defined as non-shockable without specifying Asystole or PEA<br>• Trauma aetiology<br>• Drowning	Most EMS systems are staffed with either a nurse or paramedic supported by an EMT.

© 2024 Smyth MA et al. *JAMA Network Open.*

SOS-Kanto,[12] (2017), Japan	Derivation study<br>SOS-Kanto1 TOR (n=13,291)<br>SOS-Kanto2 TOR (n=13,291)<br>SOS-Kanto3 TOR (n=13,291)<br>Validation study<br>BLS (n=13,291)**<br>Goto 2 TOR (n=13,291)	Retrospective analysis of all cardiac arrest cases occurring in Japanese Association for Acute Medicine in the Kanto Region. Excluded patient groups:<br>• Age < 18<br>• In hospital/clinic resuscitation attempt	Single tier EMS system. EMT level providers can provide defibrillation, airway adjunct insertion, IV access, IV infusion. ALS providers can perform ETI and administer adrenaline. EMS personnel cannot legally terminate resuscitation
Smits et al,[29] (2023), Netherlands	Validation study<br>ALS (n=21,808) (male)<br>ALS (n=10,772) (female)	Retrospective analysis of ARREST, SRCR & DANCAR registries. Excluded patient groups:<br>• Age < 18<br>• Presumed non-cardiac aetiology<br>• Gender data missing	ALS EMS system
Verbeek et al,[2] (2002), Canada	Derivation study<br>BLS TOR (n=622)	Retrospective analysis of all cardiac arrest cases. Excluded patient groups:<br>• Obviously deceased (rigor mortis, lividity, decomposition, decapitation)<br>• Trauma, drowning, drug overdose<br>• Received ACLS care<br>• Age < 18<br>• DNACPR	Tiered EMS system. Fire first response. Closest ambulance assigned, if BLS resource is nearest an ALS ambulance is additionally assigned provided their response time is less than 8 minutes. BLS level providers can provide semi-automatic defibrillation and King airway, while ALS providers can perform manual defibrillation, ETI, IV/IO access and administer ACLS medications
Verhaert et al,[30] (2016), Netherlands	Validation study<br>ALS TOR (n=588)	Retrospective analysis of all cardiac arrest cases in the Nijmegen region. Excluded patient groups:<br>• Age <18 years<br>• Trauma aetiology (including hanging and drowning)<br>• DNACPR order or terminal illness	Tiered response that may or may not include police or fire first response. ALS paramedic response that included option to deploy mechanical CPR (LUCAS).

© 2024 Smyth MA et al. *JAMA Network Open.*

Yates et al,[42] (2018), UK	Validation study<br>uTOR (n=220)	Retrospective analysis of all cardiac arrest cases transported to 3 hospitals in 1 city. Included cases were those where ROSC had not been achieved and the patient did not meet the UK criteria for termination of resuscitation by paramedics. There were no excluded patient groups.	Single tier ALS EMS system.
Yoon et al,[7] (2019), Korea	Derivation study<br>KoCARC 1 TOR (n=4,360)<br>KoCARC 2 TOR (n=4,360)<br>KoCARC 3 TOR (n=4,360)<br>Validation study<br>BLS (n=4,360)**	Retrospective analysis of the KoCARC database. Excluded patient groups:<br>• Age <18<br>• Obviously deceased (e.g. rigor mortis or dependent lividity)<br>• Terminal illnesses / under hospice care / DNACPR<br>• Pregnant<br>• Non-cardiac aetiology<br>• Poisoning<br>• Primary respiratory arrest<br>• Asphyxia (including hanging)<br>• Drowning<br>• Trauma (including electrocution)	Single tier EMS system. EMS personnel perform automated external defibrillation and intubation. Intravenous adrenaline is administered on medical instruction.

TOR - termination of resuscitation, ROSC – return of spontaneous circulation, ** - these studies incorrectly report efficacy of BLS TOR; they should have reported efficacy of the uTOR as the participating EMS providers functioned above BLS level, VF – ventricular fibrillation, BLS – basic life support, ALS – advanced life support, OHCA – out of hospital cardiac arrest, EMS – Emergency Medical Services, ED – Emergency Department, EMT – Emergency Medical Technician, AED – automated external defibrillator, LMA, laryngeal mask airway, IV – intravenous, IO – intraosseous, ETI – endotracheal intubation, ACLS – advanced cardiac life support, tCPA – traumatic cardiopulmonary arrest, NAEMSP – National Association of Emergency Medical Services Physicians, DNAR – do not attempt resuscitation order,

© 2024 Smyth MA et al. *JAMA Network Open.*

eTable 11. Description of Termination of Resuscitation (TOR) Rules

TOR Rule	Criteria to stop resuscitation	Exclusions
ALS	• Not witnessed by bystander **AND**<br>• No bystander CPR **AND**<br>• Not witnessed by EMS **AND**<br>• No shocks delivered (PAD or EMS) **AND**<br>• No ROSC at any time prior to transport	• Non-cardiac aetiology (including trauma, drowning, poisoning, hypothermia)<br>• Obviously deceased (rigor mortis, lividity, decomposition, decapitation)<br>• Age < 16<br>• DNACPR
BLS	• Not witnessed by EMS **AND**<br>• No shocks delivered (PAD or EMS) **AND**<br>• No ROSC at any time prior to transport (after 3rd cycle)	• Obviously deceased (rigor mortis, lividity, decomposition, decapitation)<br>• Trauma, drowning, drug overdose<br>• Received ACLS care<br>• Age < 18<br>• DNACPR
ERC	• Non-shockable rhythm **AND**<br>• Reversible causes addressed **AND**<br>• 20 minutes of asystole	• Obviously deceased (decapitation, hemicorporectomy, prolonged submersion, incineration, rigor mortis, dependent lividity and foetal maceration).
Globber 1	• Not witnessed **AND**<br>• Non-shockable rhythm **AND**<br>• Age >= 80	• Not defined
Goto 1	• Asystole **AND**<br>• Unwitnessed **AND**<br>• No bystander CPR **AND**<br>• Age >= 81 **AND**<br>• EMS resuscitation >14 min	• EMS witnessed<br>• Age < 18
Goto 2	• Not witnessed by bystander **AND**<br>• Non-shockable **AND**	• EMS witnessed<br>• Age < 18

	• No pre-hospital ROSC	
Haukoos 1	• Not witnessed **OR**<br>• Response time > 6 min **OR**<br>• Nursing home & non-shockable	• Age < 18<br>• Trauma
Helsinki	*Asystole:*<br>• Unwitnessed **OR**<br>• Ambulance response time > 10 min **OR**<br>• No ROSC despite 20 min of ALS.<br>*Witnessed PEA:*<br>• Ambulance response time > 15 min **OR**<br>• No ROSC despite 20 min of ALS.<br>*Unwitnessed PEA:*<br>• Ambulance response time > 15 min **OR**<br>• No ROSC despite 10 min of ALS.	• Hypothermia<br>• Drowning<br>• Penetrating trauma
Jabre	• Not witnessed by EMS **AND**<br>• Non-shockable initial cardiac rhythm **AND**<br>• no ROSC before receipt of a third 1-mg epinephrine dose.	• Age < 18<br>• Terminal illness<br>• DNACPR<br>• Non-cardiac aetiology
KoCARC 1	• Not witnessed by EMS **AND**<br>• Asystole **AND**<br>• No shocks delivered **AND**<br>• No ROSC at any time	• Age < 18<br>• Cut between head and body (believed to imply decapitation)<br>• Corruption (believed to imply hemicorporectomy)

© 2024 Smyth MA et al. *JAMA Network Open.*

		• Rigor mortis
KoCARC 2	• Not witnessed by EMS **AND**<br>• Age > 60 **AND**<br>• No shocks delivered **AND**<br>• No ROSC at any time	• Age < 18<br>• Cut between head and body (believed to imply decapitation)<br>• Corruption (believed to imply hemicorporectomy)<br>• Rigor mortis
KoCARC 3	• Not witnessed by EMS **AND**<br>• Asystole **AND**<br>• Age > 60 **AND**<br>• No shocks delivered **AND**<br>• No ROSC at any time	• Age < 18<br>• Cut between head and body (believed to imply decapitation)<br>• Corruption (believed to imply hemicorporectomy)<br>• Rigor mortis
KoCARC 4	• Not witnessed by bystander **AND**<br>• Asystole **AND**<br>• No ROSC at any time	• Age <18<br>• Obviously deceased (e.g. rigor mortis or dependent lividity)<br>• Terminal illnesses / under hospice care / DNACPR<br>• Pregnant<br>• Non-cardiac aetiology<br>• Trauma (including electrocution)<br>• Poisoning<br>• Primary respiratory arrest<br>• Drowning<br>• asphyxia (including hanging)
KoCARC 5	• Not witnessed by bystander **AND**<br>• No ROSC at any time **AND**<br>• Asystole on arrival at ED	• Age <18<br>• Obviously deceased (e.g. rigor mortis or dependent lividity)<br>• Terminal illnesses / under hospice care / DNACPR<br>• Pregnant

© 2024 Smyth MA et al. *JAMA Network Open.*

		• Non-cardiac aetiology<br>• Poisoning<br>• Primary respiratory arrest<br>• Asphyxia (including hanging)<br>• Drowning<br>• Trauma (including electrocution)
Marsden	• Non-shockable rhythm on arrival **AND**<br>• No bystander CPR in preceding 15 min **AND**<br>• Not excluded patient group **AND**<br>• No ROSC or shockable rhythm after 1 min CPR **AND**<br>• Continuous asystole for 10 sec	• Age < 18<br>• Pregnant<br>• Poisoning or overdose<br>• Hypothermia<br>• Drowning
MIEMS (medical)	• Age <18<br>• EMS scene time >30min<br>• Initial non-shockable rhythm<br>• EtCO2<15mmHg	• Age>18<br>• Trauma
MIEMS (trauma)	• Age <18 or 15<br>• EMS scene time >10min<br>• Initial non-shockable rhythm<br>• EtCO2<15mmHg<br>•	• Age >18<br>• Medical
NAEMSP	• Received > 20 min ALS (definitive airway, defibrillation, IV ACLS drug therapy) **AND**<br>• Remains non-shockable **AND**<br>• No ROSC at any time	• Age < 18<br>• Trauma<br>• Hypothermia<br>• Drowning
No ROSC	• No ROSC at any time	• Persistent VF<br>• Age < 18<br>• Trauma<br>• Hypothermia<br>• Primary respiratory aetiology<br>• Overdose

© 2024 Smyth MA et al. *JAMA Network Open.*

PEA	•	•
Petrie	• Asystole **AND**<br>• Response time > 8 min	• Non-cardiac aetiology (inc trauma, drowning, poisoning, hypothermia)<br>• Obviously deceased (rigor mortis, lividity, decomposition, decapitation)<br>• Age < 16<br>• DNACPR
Shibahashi 1	• Non-shockable rhythm **AND**<br>• Not witnessed **AND**<br>• Age >=73	• Age > 120<br>• EMS physician team
Shibahashi 3	•	•
SOS Kanto 1	• Not witnessed by bystander **AND**<br>• Non-shockable in field **AND**<br>• Non-shockable on arrival at ED	• Age < 18<br>• Obvious signs of death (e.g. decapitation, incineration, decomposition, rigor mortis, or dependent lividity)
SOS Kanto 2	• Not witnessed by bystander **AND**<br>• Non-shockable in field **AND**<br>• Asystole on arrival at ED	• Age < 18<br>• Obvious signs of death (e.g. decapitation, incineration, decomposition, rigor mortis, or dependent lividity)
SOS Kanto 3	• Not witnessed by bystander **AND**<br>• Asystole in field **AND**<br>• Asystole on arrival at ED	• Age < 18<br>• Obvious signs of death (e.g. decapitation, incineration, decomposition, rigor mortis, or dependent lividity)
tCPA	• Blunt trauma **AND**<br>• Asystole	• Age < 18<br>• Obviously deceased (e.g. decapitation or rigour mortis)

© 2024 Smyth MA et al. *JAMA Network Open.*

		• DNACPR<br>• Any medical cause<br>• Penetrating trauma
uTOR	• Not witnessed by EMS **AND**<br>• No shocks delivered (PAD or EMS) **AND**<br>• No ROSC at any time prior to transport (after 3rd cycle)	• Age < 18<br>• Obviously deceased (rigor mortis, lividity, decomposition, decapitation)<br>• Trauma, drowning, drug overdose<br>• DNACPR

© 2024 Smyth MA et al. *JAMA Network Open.*

eFigure 1. QUADAS-2 Summary Risk of Bias and Applicability

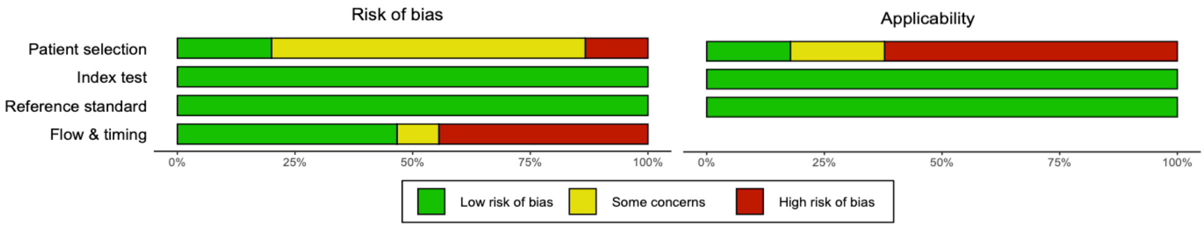

© 2024 Smyth MA et al. *JAMA Network Open.*

eFigure 2. Study Risk of Bias

Author year	Risk of bias: Patient Selection	Risk of bias: Index Test	Risk of bias: Reference Standard	Risk of bias: Flow and Timing	Applicability: Patient Selection	Applicability: Index Test	Applicability: Reference Standard
Bonnin et al,[14] (1993)	?	+	+	+	×	+	+
Cheong et al,[16] (2016)	?	+	+	+	×	+	+
Chiang et al,[17] (2015)	?	+	+	+	?	+	+
Chiang et al,[15] (2016)	?	+	+	+	×	+	+
Cone et al,[37] (2005)	?	+	+	×	×	+	+
Diskin et al,[18] (2014)	?	+	+	×	×	+	+
Drennan et al,[39] (2014)	?	+	+	×	×	+	+
Fukada et al,[19] (2014)	?	+	+	+	+	+	+
Glober et al,[3] (2019)	+	+	+	×	×	+	+
Glober et al,[34] (2020)	+	+	+	×	×	+	+
Goto et al,[4] (2019)	+	+	+	+	+	+	+
Grunau et al,[40] (2017)	?	+	+	×	×	+	+
Grunau et al,[38] (2019)	?	+	+	×	×	+	+
Harris et al,[36] (2021)	+	+	+	+	+	+	+
Haukoos et al,[5] (2004)	?	+	+	?	?	+	+
House et al,[10] (2018)	+	+	+	×	×	+	+
Hreinsson et al,[20] (2020)	?	+	+	×	×	+	+
Hsu et al,[21] (2022)	+	+	+	×	+	+	+
Jabre et al,[6] (2016)	×	+	+	×	×	+	+
Jordan et al,[41] (2017)	?	+	+	×	×	+	+
Kajino et al,[22] (2013)	?	+	+	+	×	+	+
Kashiura et al,[23] (2016)	?	+	+	+	+	+	+
Kim et al,[31] (2015)	?	+	+	+	×	+	+
Lee et al,[8] (2019)	?	+	+	+	?	+	+
Lin et al,[24] (2022)	?	+	+	+	?	+	+
Marsden et al,[9] (1995)	?	+	+	×	×	+	+
Matsui et al,[25] (2023)	+	+	+	+	+	+	+
Morrison et al,[1] (2007)	?	+	+	?	?	+	+
Morrison et al,[26] (2009)	?	+	+	×	×	+	+
Morrison et al,[43] (2014)	?	+	+	×	×	+	+
Ong et al,[32] (2006)	?	+	+	×	×	+	+
Ong et al,[33] (2007)	+	+	+	+	+	+	+
Park et al,[35] (2023)	×	+	+	×	×	+	+
Petrie et al,[11] (2001)	?	+	+	×	×	+	+

© 2024 Smyth MA et al. *JAMA Network Open.*

Sasson et al,[27] (2008)	?	+	+	×	×	+	+
Shibahashi et al,[13] (2020)	×	+	+	×	×	+	+
Skrifvars et al,[28] (2010)	×	+	+	×	×	+	+
SOS-Kanto,[12] (2017)	+	+	+	+	+	+	+
Smits et al,[29] (2023)	×	+	+	×	×	+	+
Verbeek et al,[2] (2002)	?	+	+	+	?	+	+
Verhaert et al,[30] (2016)	?	+	+	×	×	+	+
Yates et al,[42] (2018)	×	+	+	+	×	+	+
Yoon et al,[7] (2019)	?	+	+	+	?	+	+

× high risk. ? risk unclear + low risk

eFigure 3. Deeks Funnel Plot Asymmetry Test

BLS TOR rule

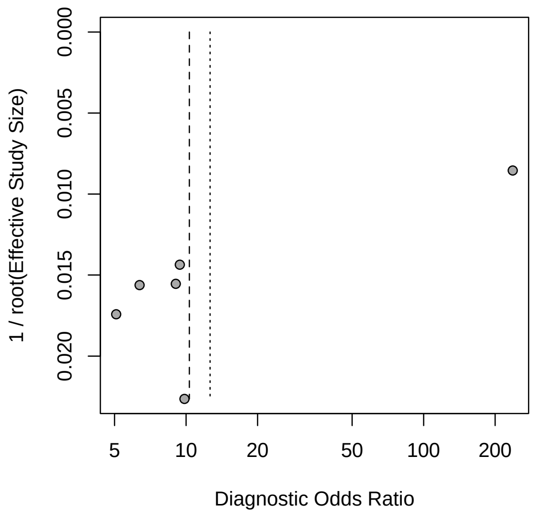

ALS TOR rule

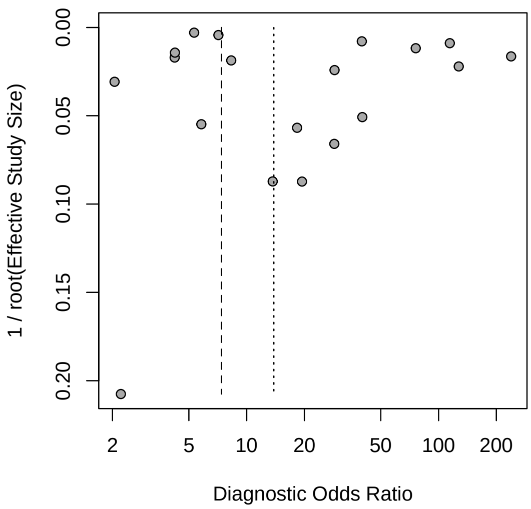

uTOR rule

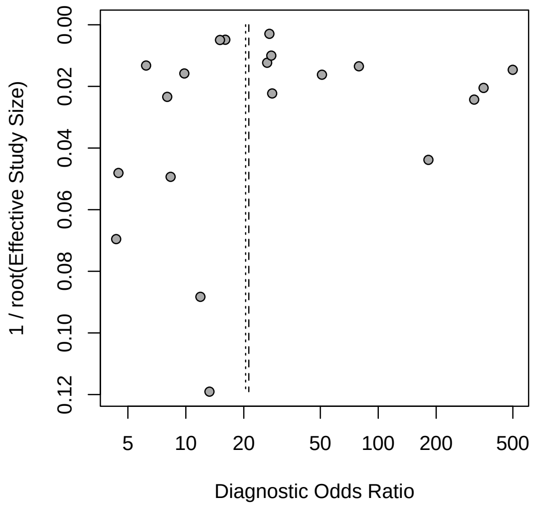

© 2024 Smyth MA et al. *JAMA Network Open.*

eFigure 4. Bivariate Summary Receiver Operating Characteristic (SROC) Curves

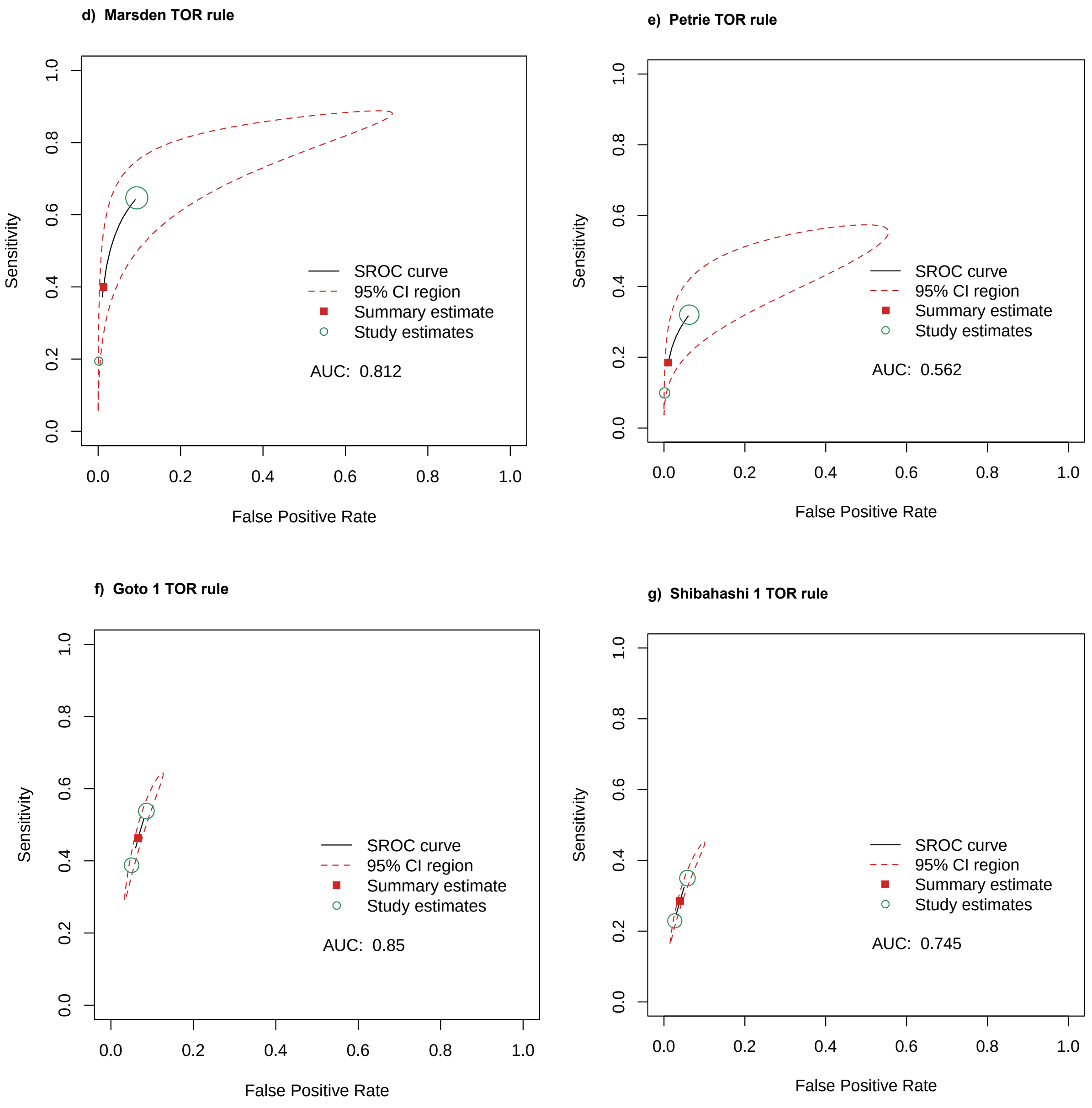

© 2024 Smyth MA et al. *JAMA Network Open.*

eReferences

1. Morrison LJ, Verbeek PR, Vermeulen MJ, et al. Derivation and evaluation of a termination of resuscitation clinical prediction rule for advanced life support providers. Resuscitation 2007;74:266-75.
2. Verbeek PR, Vermeulen MJ, Ali FH, Messenger DW, Summers J, Morrison LJ. Derivation of a termination-of-resuscitation guideline for emergency medical technicians using automated external defibrillators. Academic Emergency Medicine 2002;9:671-8.
3. Glober NK, Tainter CR, Abramson TM, Staats K, Gilbert G, Kim D. A simple decision rule predicts futile resuscitation of out-of-hospital cardiac arrest. Resuscitation 2019;142:8-13.
4. Goto Y, Funada A, Maeda T, Okada H, Goto Y. Field termination-of-resuscitation rule for refractory out-of-hospital cardiac arrests in Japan. J Cardiol 2019;73:240-6.
5. Haukoos JS, Lewis RJ, Niemann JT. Prediction rules for estimating neurologic outcome following out-of-hospital cardiac arrest. Resuscitation 2004;63:145-55.
6. Jabre P, Bougouin W, Dumas F, et al. Early Identification of Patients With Out-of-Hospital Cardiac Arrest With No Chance of Survival and Consideration for Organ Donation. Annals of Internal Medicine 2016;165:770-8.
7. Yoon JC, Kim Y-J, Ahn S, et al. Factors for modifying the termination of resuscitation rule in out-of-hospital cardiac arrest. Am Heart J 2019;213:73-80.
8. Lee DE, Lee MJ, Ahn JY, et al. New Termination-of-Resuscitation Models and Prognostication in Out-of-Hospital Cardiac Arrest Using Electrocardiogram Rhythms Documented in the Field and the Emergency Department. J Korean Med Sci 2019;34:e134.
9. Marsden AK, Ng GA, Dalziel K, Cobbe SM. When is it futile for ambulance personnel to initiate cardiopulmonary resuscitation? BMJ 1995;311:49-51.
10. House M, Gray J, McMeekin P. Reducing the futile transportation of out-of-hospital cardiac arrests: a retrospective validation. Br Paramed J 2018;3:1-6.
11. Petrie DA, De Maio V, Stiell IG, Dreyer J, Martin M, O'Brien J A. Factors affecting survival after prehospital asystolic cardiac arrest in a Basic Life Support-Defibrillation system. Cjem 2001;3:186-92.
12. Group S-KS. A New Rule for Terminating Resuscitation of Out-of-Hospital Cardiac Arrest Patients in Japan: A Prospective Study. Journal of Emergency Medicine 2017;53:345-52.
13. Shibahashi K, Sugiyama K, Hamabe Y. A potential termination of resuscitation rule for EMS to implement in the field for out-of-hospital cardiac arrest: An observational cohort study. Resuscitation 2018;130:28-32.
14. Bonnin MJ, Pepe PE, Kimball KT, Clark PS, Jr. Distinct criteria for termination of resuscitation in the out-of-hospital setting. JAMA 1993;270:1457-62.
15. Chiang WC, Huang YS, Hsu SH, et al. Performance of a simplified termination of resuscitation rule for adult traumatic cardiopulmonary arrest in the prehospital setting. Emerg Med J 2017;34:39-45.
16. Cheong RW, Li H, Doctor NE, et al. Termination of Resuscitation Rules to Predict Neurological Outcomes in Out-of-Hospital Cardiac Arrest for an Intermediate Life Support Prehospital System. Prehosp Emerg Care 2016;20:623-9.
17. Chiang WC, Ko PC, Chang AM, et al. Predictive performance of universal termination of resuscitation rules in an Asian community: are they accurate enough? Emergency Medicine Journal 2015;32:318-23.
18. Diskin FJ, Camp-Rogers T, Peberdy MA, Ornato JP, Kurz MC. External validation of termination of resuscitation guidelines in the setting of intra-arrest cold saline, mechanical CPR, and comprehensive post resuscitation care. Resuscitation 2014;85:910-4.
19. Fukuda T, Ohashi N, Matsubara T, et al. Applicability of the prehospital termination of resuscitation rule in an area dense with hospitals in Tokyo: a single-center, retrospective, observational study: is the pre hospital TOR rule applicable in Tokyo? Am J Emerg Med 2014;32:144-9.
20. Hreinsson JP, Thorvaldsson AP, Magnusson V, Fridriksson BT, Libungan BG, Karason S. Identifying out-of-hospital cardiac arrest patients with no chance of survival: An independent validation of prediction rules. Resuscitation 2020;146:19-25.

© 2024 Smyth MA et al. *JAMA Network Open.*

21. Hsu SH, Sun JT, Huang EP, et al. The predictive performance of current termination-of-resuscitation rules in patients following out-of-hospital cardiac arrest in Asian countries: A cross-sectional multicentre study. PLoS One 2022;17:e0270986.
22. Kajino K, Kitamura T, Iwami T, et al. Current termination of resuscitation (TOR) guidelines predict neurologically favorable outcome in Japan. Resuscitation 2013;84:54-9.
23. Kashiura M, Hamabe Y, Akashi A, et al. Applying the termination of resuscitation rules to out-of-hospital cardiac arrests of both cardiac and non-cardiac etiologies: a prospective cohort study. Crit Care 2016;20:49.
24. Lin Y-Y, Lai Y-Y, Chang H-C, et al. Predictive performances of ALS and BLS termination of resuscitation rules in out-of-hospital cardiac arrest for different resuscitation protocols. BMC emergency medicine 2022;22:53.
25. Matsui S, Kitamura T, Kurosawa H, et al. Application of adult prehospital resuscitation rules to pediatric out of hospital cardiac arrest. Resuscitation 2023;184:109684.
26. Morrison LJ, Verbeek PR, Zhan C, Kiss A, Allan KS. Validation of a universal prehospital termination of resuscitation clinical prediction rule for advanced and basic life support providers. Resuscitation 2009;80:324-8.
27. Sasson C, Hegg AJ, Macy M, et al. Prehospital termination of resuscitation in cases of refractory out-of-hospital cardiac arrest. JAMA 2008;300:1432-8.
28. Skrifvars MB, Vayrynen T, Kuisma M, et al. Comparison of Helsinki and European Resuscitation Council "do not attempt to resuscitate" guidelines, and a termination of resuscitation clinical prediction rule for out-of-hospital cardiac arrest patients found in asystole or pulseless electrical activity. Resuscitation 2010;81:679-84.
29. Smits RLA, Sodergren STF, van Schuppen H, et al. Termination of resuscitation in out-of-hospital cardiac arrest in women and men: An ESCAPE-NET project. Resuscitation 2023;185:109721.
30. Verhaert DV, Bonnes JL, Nas J, et al. Termination of resuscitation in the prehospital setting: A comparison of decisions in clinical practice vs. recommendations of a termination rule. Resuscitation 2016;100:60-5.
31. Kim TH, Shin SD, Kim YJ, Kim CH, Kim JE. The scene time interval and basic life support termination of resuscitation rule in adult out-of-hospital cardiac arrest. J Korean Med Sci 2015;30:104-9.
32. Ong MEH, Jaffey J, Stiell I, Nesbitt L. Comparison of termination-of-resuscitation guidelines for basic life support: Defibrillator providers in out-of-hospital cardiac arrest. Annals of Emergency Medicine 2006;47:337-43.
33. Ong ME, Tan EH, Ng FS, et al. Comparison of termination-of-resuscitation guidelines for out-of-hospital cardiac arrest in Singapore EMS. Resuscitation 2007;75:244-51.
34. Glober NK, Lardaro T, Christopher S, Tainter CR, Weinstein E, Kim D. Validation of the NUE Rule to Predict Futile Resuscitation of Out-of-Hospital Cardiac Arrest. Prehospital emergency care : official journal of the National Association of EMS Physicians and the National Association of State EMS Directors 2020:1-6.
35. Park SY, Lim D, Ryu JH, Kim YH, Choi B, Kim SH. Modification of termination of resuscitation rule with compression time interval in South Korea. Scientific reports 2023;13:1403.
36. Harris MI, Crowe RP, Anders J, D'Acunto S, Adelgais KM, Fishe J. Applying a set of termination of resuscitation criteria to paediatric out-of-hospital cardiac arrest. Resuscitation 2021;169:175-81.
37. Cone DC, Bailey ED, Spackman AB. The safety of a field termination-of-resuscitation protocol. Prehospital Emergency Care 2005;9:276-81.
38. Grunau B, Scheuermeyer F, Kawano T, et al. North American validation of the Bokutoh criteria for withholding professional resuscitation in non-traumatic out-of-hospital cardiac arrest. Resuscitation 2019;135:51-6.
39. Drennan IR, Lin S, Sidalak DE, Morrison LJ. Survival rates in out-of-hospital cardiac arrest patients transported without prehospital return of spontaneous circulation: an observational cohort study. Resuscitation 2014;85:1488-93.

© 2024 Smyth MA et al. *JAMA Network Open.*

40. Grunau B, Taylor J, Scheuermeyer FX, et al. External Validation of the Universal Termination of Resuscitation Rule for Out-of-Hospital Cardiac Arrest in British Columbia. Annals of Emergency Medicine 2017;70:374-81.e1.
41. Jordan MR, O'Keefe MF, Weiss D, Cubberley CW, MacLean CD, Wolfson DL. Implementation of the universal BLS termination of resuscitation rule in a rural EMS system. Resuscitation 2017;118:75-81.
42. Yates EJ, Schmidbauer S, Smyth AM, et al. Out-of-hospital cardiac arrest termination of resuscitation with ongoing CPR: An observational study. Resuscitation 2018;130:21-7.
43. Morrison LJ, Eby D, Veigas PV, et al. Implementation trial of the basic life support termination of resuscitation rule: reducing the transport of futile out-of-hospital cardiac arrests. Resuscitation 2014;85:486-91.

© 2024 Smyth MA et al. *JAMA Network Open.*
