# Supplementary material for: Termination of Resuscitation Rules and Survival Among Patients With Out-of-Hospital Cardiac Arrest: A Systematic Review and Meta-Analysis
Source: JAMA Netw Open. 2024 Jul 3;7(7):e2420040. doi: 10.1001/jamanetworkopen.2024.20040 (PMC11222995; doi:10.1001/jamanetworkopen.2024.20040)
Supplement: Supplement 2. — Data Sharing Statement [file jamanetwopen-e2420040-s002.pdf]

Data Sharing Statement

Smyth. Termination of Resuscitation Rules and Survival Among Patients With Out-of-Hospital Cardiac Arrest. *JAMA Netw Open*. Published July 03, 2024.
doi:10.1001/jamanetworkopen.2024.20040

Data
Data available: Yes
Data types: Other (please specify)
Additional Information: contingency table data for each included study
How to access data: m.a.smyth@warwick.ac.uk
When available: With publication

Supporting Documents
Document types: None

Additional Information
Who can access the data: researchers whose proposed use of the data has been approved
Types of analyses: research purposes
Mechanisms of data availability: after approval of a proposal
